# Macromolecule–Nanoparticle-Based Hybrid Materials for Biosensor Applications

**DOI:** 10.3390/bios14060277

**Published:** 2024-05-28

**Authors:** Giddaerappa Kuntoji, Naseem Kousar, Shivalingayya Gaddimath, Lokesh Koodlur Sannegowda

**Affiliations:** Department of Studies in Chemistry, Vijayanagara Sri Krishnadevaraya University, Jnanasagara, Vinayakanagara, Ballari 583105, India; giddaerappa1234@gmail.com (G.K.); naseem.kousar9999@gmail.com (N.K.); shivalingayyagaddimath@gmail.com (S.G.)

**Keywords:** biosensor, biomolecules, macromolecules, macrocycles, limit of detection, sensitivity, limit of quantification, glucose, hydrogen peroxide, uric acid, dopamine, cholesterol, cancer cells, amino acids

## Abstract

Biosensors function as sophisticated devices, converting biochemical reactions into electrical signals. Contemporary emphasis on developing biosensor devices with refined sensitivity and selectivity is critical due to their extensive functional capabilities. However, a significant challenge lies in the binding affinity of biosensors to biomolecules, requiring adept conversion and amplification of interactions into various signal modalities like electrical, optical, gravimetric, and electrochemical outputs. Overcoming challenges associated with sensitivity, detection limits, response time, reproducibility, and stability is essential for efficient biosensor creation. The central aspect of the fabrication of any biosensor is focused towards forming an effective interface between the analyte electrode which significantly influences the overall biosensor quality. Polymers and macromolecular systems are favored for their distinct properties and versatile applications. Enhancing the properties and conductivity of these systems can be achieved through incorporating nanoparticles or carbonaceous moieties. Hybrid composite materials, possessing a unique combination of attributes like advanced sensitivity, selectivity, thermal stability, mechanical flexibility, biocompatibility, and tunable electrical properties, emerge as promising candidates for biosensor applications. In addition, this approach enhances the electrochemical response, signal amplification, and stability of fabricated biosensors, contributing to their effectiveness. This review predominantly explores recent advancements in utilizing macrocyclic and macromolecular conjugated systems, such as phthalocyanines, porphyrins, polymers, etc. and their hybrids, with a specific focus on signal amplification in biosensors. It comprehensively covers synthetic strategies, properties, working mechanisms, and the potential of these systems for detecting biomolecules like glucose, hydrogen peroxide, uric acid, ascorbic acid, dopamine, cholesterol, amino acids, and cancer cells. Furthermore, this review delves into the progress made, elucidating the mechanisms responsible for signal amplification. The Conclusion addresses the challenges and future directions of macromolecule-based hybrids in biosensor applications, providing a concise overview of this evolving field. The narrative emphasizes the importance of biosensor technology advancement, illustrating the role of smart design and material enhancement in improving performance across various domains.

## 1. Introduction

Sensors are essential tools for detecting and measuring a variety of physical and chemical phenomena, such as motion, pressure, heat, and light [1]. With time, these devices have undergone substantial evolution, incorporating a wide range of features and being used in industries including automotive, industrial, medicinal, agricultural, aerospace, and defense [2]. Owing to their capacity to monitor biological processes, diagnose illnesses, identify environmental contaminants, and aid in drug discovery, biomolecule or biomarker sensors (also referred to as biosensors) hold immense significance across various domains [3]. Biosensors provide significant understanding towards conditions, disease progression, and environmental health by detecting biomolecules such as proteins, deoxyribonucleic acid (DNA) [4], ribonucleic acid (RNA), enzymes, and hormones [5]. This ability is especially important for medical diagnostics, since prompt and precise biomarker identification can help with early disease diagnosis and individualized treatment strategies [6]. Furthermore, the detection of pollutants [7] and contaminants [8] is made easier with the help of sensing and monitoring, which is essential for environmental sustainability and public health [9].

Biomolecules are the basic constituents of life, playing critical roles in various biological processes that are essential to the proper functioning of living organisms [10]. These molecules include a wide variety of compounds, such as proteins, lipids, carbohydrates [11], nucleic acids, and metabolites [12]. They serve as energy sources, transmit genetic information, catalyze biochemical reactions, and provide structural support. Specifically, proteins are multifunctional molecules that are essential for many biological processes, including structural support, cellular signaling, and enzyme catalysis [13,14]. Nucleic acids, which include DNA and RNA, encode genetic information and play central role in the replication, transcription, and translation of genetic material [15,16]. Lipids are molecules that store energy, function as signaling molecules, and make up the structural elements of cell membranes. Carbohydrates serve as building blocks and cell surface markers, and also as energy sources [17]. Small molecules called metabolites are part of metabolic pathways and can act as substrates, intermediates, or products in various biochemical reactions [18,19]. The preservation of cellular functions and the general health of an organism depend on the delicate balance and composition of biomolecules. Small changes in their amount or composition can cause malfunction and be a factor in a number of illnesses or health problems. For example, disorders in the folding of proteins can lead to neurodegenerative conditions like Parkinson’s or Alzheimer’s [20]. Gene abnormalities or cancer can result from DNA mutations [21]. Metabolic diseases such as obesity and cardiovascular disorders may be exacerbated by dysregulation of lipid metabolism [22]. It is important to precisely identify and measure biomolecules because of their vital roles in both health and diseases [23]. Analytical tools known as biosensors use a transducer and a biological sensing element to transform a biological response into a signal that can be measured [24]. Their ability to detect biomolecules in real time, with high sensitivity and selectivity, makes them an invaluable tool in a wide range of applications, such as food safety, environmental monitoring, medical diagnosis, and biotechnology [25].

Several biosensor technologies have been developed to aid in biomolecule sensing, each operating on a different principle. One example is optical biosensors, which identify and measure analytes using the interplay between biomolecules and light [26]. They normally use a biological recognition element that has been immobilized on a surface. Analyte concentration is determined by measuring changes in light characteristics caused by target biomolecules bound to recognition elements, such as intensity, wavelength, or polarization [27]. Another example is piezoelectric biosensors, which track mass or viscoelastic property changes resulting from binding of biomolecules [28]. Usually, they use a piezoelectric substance, like quartz, whose resonance frequency is modified when biomolecules bind to its surface. The concentration of the target analytes can be found by measuring this frequency shift [29]. Surface plasmon resonance (SPR) biosensors take into account the surface plasmon resonance phenomenon, which occurs when light interacts with a metal surface in particular ways [30]. Typically, these sensors are made of a thin metal film with a biological recognition element coated on it. Shifts in the angle or intensity of reflected light can be used to detect changes in the refractive index near the surface caused by target analytes bound to recognition elements [31]. Electrochemical biosensors measure the electrical signals produced at electrode surfaces to identify biomolecule binding events [32]. Enzymes and other biological recognition elements immobilized on electrodes are frequently used. Analyte concentration can be detected by measuring changes in electrical characteristics brought about by target analytes interacting with these elements, such as current, voltage, or impedance [33]. The sensitivity, affordability, portability, biocompatibility, simplicity, and quick response of electrochemical biosensors in particular make them highly promising in detection of analytes [34]. Usually consisting of three electrodes (reference, counter, and working) [35], electrochemical biosensors detect electrical signals using methods like potentiometry, cyclic voltammetry (CV) [36], chronoamperometry (CA), differential pulse voltammetry (DPV) [37], impedimetric, and linear sweep voltammetry (LSV) [38].

The working electrode pinhole surface interacts with the analyte in the electrochemical biosensing mechanism, triggering a redox reaction [39]. By adding different conducting noble materials, like platinum (Pt) and gold (Au) to electrode surfaces, researchers have improved the performance of biosensors by enabling faster electron movement [40]. Currently, a variety of redox-active single element biosensors are used on the electrode surface, including metal oxides, carbonaceous materials (like graphene and carbon nanotubes), nanoparticles, and new organic compounds [41]. High sensitivity and customizable surface characteristics are provided by nanoparticle-based biosensor materials. However, they may be susceptible to aggregation and complex synthesis [42]. In microfabrication, metal oxides offer stability and compatibility [43], but they are not selective and are prone to interference [44]. The mechanical strength and conductivity of carbonaceous materials are high, but they are also susceptible to nonspecific binding and surface fouling [45]. While novel organic compounds offer a wide range of molecular structures, their stability and sensitivity may be controlled [46]. Organic-based macrocycles, such as phthalocyanines (Pcs) [47] and porphyrins [48], as well as other macromolecules like dendrimers [49], cyclodextrins [50,51,52], calixarenes, and polymers, have attracted a lot of attention lately, as a result of their dependability in signal amplification during electrochemical sensing [36]. This has opened up a lot of possibilities for the development of advanced biosensors. Due to their unique structural characteristics and superior optical qualities, Pc- and porphyrin-based aromatic macrocyclic compounds are useful as biosensor component material [53,54]. Their conjugated π-electron system [55] and planar structure allow for efficient electron transfer [56] and catalytic activity [57], which in turn allow for sensitive analyte detection [58]. Furthermore, macromolecules that provide tunable properties and biocompatibility [59,60], like polymers, dendrimers, calixarenes, and cyclodextrins, enable the selective immobilization of biomolecules and improve stability in biosensing applications [61,62]. However, these macromolecules face issues related to aggregation and nonspecific binding, limiting their effectiveness in some biosensing applications.

**Scheme 1:**
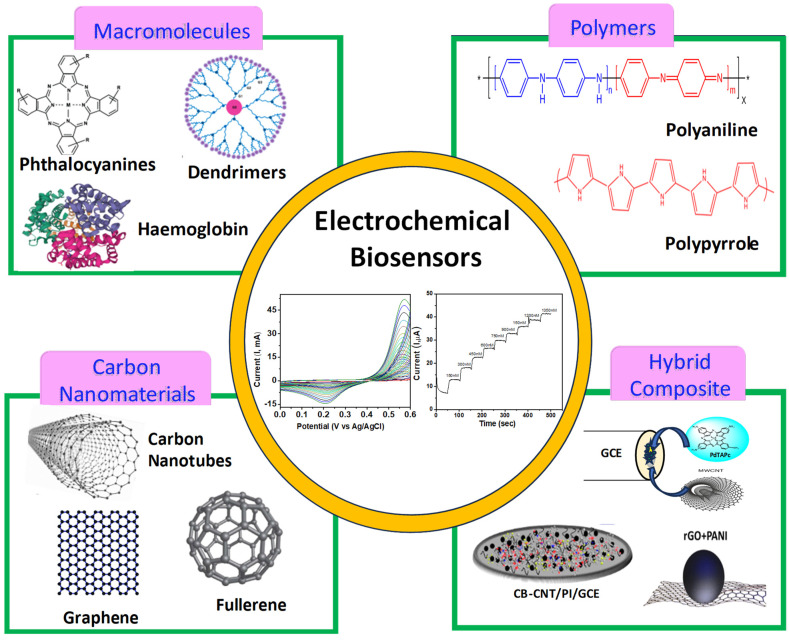
Roadmap of various macromolecules and their hybrid composites for biosensor applications.

In order to overcome the limitations of single element-based biosensors i.e., nanoparticles or carbonaceous materials and macromolecules, the integration of these organic systems with metal oxides or carbonaceous materials creates nanohybrid composites that provide a potential remedy [63,64]. Nanohybrids offer a synergistic framework and a unique combination of properties. Carbon nanomaterials (graphene, carbon nanotubes, graphene oxide, etc.) contribute to enhanced electrical conductivity and a large surface area, while macrocycles provide tenable biocompatibility, enhanced sensor responsiveness, and processability [39,65]. Furthermore, the inclusion of metal nanoparticles enhances the sensing capability of nanohybrid materials by adding significant catalytic activity, signal amplification, and distinctive optical properties [66]. These hybrid composites, addressing the limitations of aforementioned biosensors, enhance biosensor performance in terms of sensitivity, detection limit, selectivity, multifunctionality, and miniaturization (Scheme 1) [67]. However, hurdles related to aggregation, optimizing high porosity, surface area, and conductivity of hybrid composites [68], guaranteeing a prompt biosensor response by maintaining stacking arrangement for long-term stability, and achieving strong affinity for the immobilization of hybrid composites on electrode surfaces in hierarchical structures still need to be resolved [69]. Recent developments demonstrate how macrocycle-based hybrid composites can overcome these obstacles and improve the effectiveness of biosensors across a range of applications. This review notably focuses on the advancements made in sensing of several important biomolecules, including glucose, hydrogen peroxide, uric acid, ascorbic acid, dopamine, cholesterol, amino acids, and cancer cells, over the past decade, recognizing their critical role in human health. A thorough discussion ensues regarding the role of macrocyclic hybrid sensors in detecting these molecules, and the final Section examines the difficulties and potential future directions of these hybrid-based macrocycles as biosensors, suggesting the possibility of significant advancements in the field. The unique aspect of selecting these analytes for review lies in their comprehensive coverage of key biomolecules essential for understanding a wide range of physiological and pathological processes. Unlike other reviews focusing on narrow set of analytes or specific applications, this review focuses on a diverse array of molecules, facilitating a holistic exploration of sensing techniques across various disciplines. Furthermore, the inclusion of cancer cells alongside traditional biomarkers highlights the forward-thinking approach, recognizing the increasing significance of early cancer detection and personalized health care. By incorporating such a broad spectrum of analytes, this review not only provides a comprehensive overview of current sensing methodologies but also encourages interdisciplinary collaboration and innovation. Thus, it serves as a valuable resource for advancing diagnostics, therapeutics, and healthcare technologies.

## 2. Fundamentals and Parameters for Sensing of Biomolecules

**a.**  **Fundamentals of sensing of biomolecules:**
  - *Recognition Element*: Using particular interactions like antigen–antibody binding or enzyme–substrate catalysis, the recognition element—which includes aptamers, antibodies, enzymes, and molecularly imprinted polymers—interacts selectively with the target analyte to determine the specificity of the sensor.
  - *Transducer*: Transducers are available in a variety of forms, including optical (such as fluorescence and absorbance), electrochemical (such as amperometry and potentiometry), and mechanical (such as piezoelectric) sensors. They function by changing the altered physical or chemical properties of the sensor to convert the recognition and binding events into measurable signals.
  - *Signal Processing*: In order to extract meaningful information about the target analyte while improving the signal-to-noise ratio, eliminating interference, and extracting pertinent information, signal processing techniques like amplification, filtering, and data analysis are used to manipulate and analyze the raw sensor signal.
  - *Detection Mechanism*: The process by which the target biomolecule binds to the recognition element is known as the detection mechanism. Depending on the type of transducer used, this process involves changes in optical, electrochemical, or mechanical properties that allow the sensor to determine the presence or concentration of the target analyte based on the strength or type of signal that is generated.
  - *Calibration and Validation*: Validation ensures that sensor measurements are accurate, consistent, and reliable under a range of conditions, confirming the sensor’s suitability for the intended use. Calibration establishes the relationship between the concentration of the target analyte and the sensor output.
  - *Miniaturization and Integration*: Miniaturizing sensor parts and incorporating them into implantable or portable devices are key developments in sensing technology. Integration increases usability and accessibility across a range of applications, while miniaturization improves sensitivity, selectivity, and response time.
**b.**  **Parameters of sensing of biomolecules:**

A physical quantity or property is detected or measured during the sensing process, and when designing and evaluating sensors, a number of factors are taken into account. These parameters aid in ensuring dependable and accurate sensing. A few typical sensing parameters are:

- *Sensitivity*: The term “sensitivity” describes a sensor’s capacity to recognize variations in the quantity being measured. It shows the extent to which a change in the input stimulus alters the sensor’s output. Minor changes in the measured parameter can be retrieved through a highly sensitive sensor.
- *Selectivity/Specificity*: The term “selectivity” or “specificity” describes a sensor’s capacity to react exclusively to the target analyte or element of interest, disregarding interference from other substances or outside variables.
- *Resolution:* The smallest change in the measured quantity that the sensor can distinguish is referred to as resolution. It stands for the smallest input increment that can cause the sensor’s output to a noticeable change.
- *Linearity*: Plotting the sensor response against the actual input values and evaluating its linearity determines how well the response implies a straight line. Throughout its measurement range, a linear sensor shows a constant relationship between the input and output.
- *Range*: The range denotes the lowest and maximum values of the parameter being measured that the sensor is capable of precisely detecting or measuring. It outlines the range in which the sensor functions optimally.
- *Response Time*: The duration taken by the sensor to recognize and respond to alterations in the input stimulus is measured as its response time. It shows how accurate the sensor can be when measuring under different conditions.
- *Stability*: The ability of the sensor to retain its calibration and performance characteristics over time is referred to as stability. In continuous operation, a stable sensor shows very little drift or output variations.
- *Limit of Detection (LOD)*: A sensor can reliably detect the lowest concentration or smallest amount of an analyte or parameter above the background noise at the limit of detection. The lower limit of the sensor’s measurable range is defined by it, and statistical techniques like signal-to-noise ratio analysis are frequently used to determine LOD. The LOD is particularly significant for applications that need to detect small changes in the measured quantity or low concentrations. A lower LOD indicates a sensor’s increased sensitivity and ability to detect trace amounts of the target analyte amidst noise or interference.

The above parameters are critical in the design, evaluation, and selection of sensors for a variety of applications, ensuring maximal performance and reliability.

## 3. Fabrication of Working Electrode towards Sensing of Biomolecules

In order to ensure effective charge transfer in biosensors, fabrication techniques play a critical role in optimizing the interface between the working electrode and biomolecules [70]. These adaptable methods, which are designed for particular biosensing applications, include photolithography, screen printing, and thin film deposition, among others [71,72]. Thin film deposition creates a homogeneous surface for biomolecule immobilization by precisely depositing conductive materials, like carbon, platinum, or gold, onto a substrate [73,74]. With screen printing, which is quick and affordable, conductive ink is applied to a substrate using a mesh screen stencil to produce high-resolution electrode patterns [75,76]. By employing light-sensitive photoresist for electrode patterning, photolithography allows for precise micron-level accuracy through selective etching [64,77]. Furthermore, customized electrode properties like morphology, size, and surface chemistry can be obtained through electrochemical deposition, inkjet printing, and microfabrication techniques like soft lithography and microcontact printing [78,79]. Self-assembled monolayers (SAMs) create a stable interface for immobilizing biomolecules, and the addition of nanomaterials improves the kinetics of charge transfer and expands the surface area that is available for binding biomolecules [80,81].

**Scheme 2:**
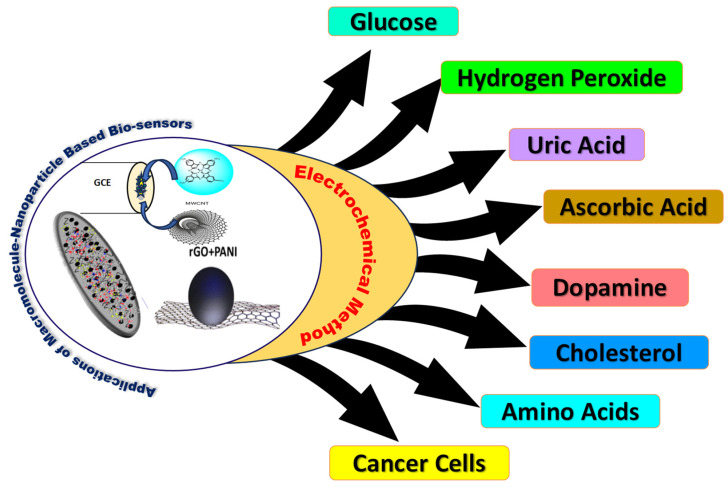
Representation of macromolecule-based hybrid composites for the detection of various biomolecules using electrochemical techniques.

Additionally, the immobilization of macrocycles on the electrode surface is mediated by non-covalent interactions such as van der Waals, ionic, and hydrophobic interactions, which are generally accomplished through straightforward physical adsorption via drop casting [82,83]. On the other hand, catalytic activity is not significant because of the poor conductivity of the macrocycle and weaker physical interactions. In order to create hybrid composites for electrochemical biosensors, the combination of macrocycles and carbon nanoparticles has recently attracted interest. By adding electrocatalytically active macrocycles to the highly conductive surface of carbon nanoparticles (CNPs), the aromatic sp^2^ hybridized carbon network and macrocycles interact via π-π attractions which leads to improvement in conductivity resulting in improved charge transfer kinetics [84]. Designing working electrodes with such hybrid composites provide appealing benefits such as increased surface area, faster electron transfer kinetics, and increased conductivity for electrochemical analyte conversion, allowing for a greater number of biomolecules interactions [70,85]. Furthermore, microfabrication techniques, such as microfluidics, enable the integration of miniaturized electrodes with fluid handling systems, thereby improving biosensor functionality and portability for optimal sensitivity, selectivity, and reliability across a wide range of applications. Scheme 2 presents the summary of sensors discussed for different biomolecules in this review paper.

## 4. Electrochemical Sensing of Biomolecules

### 4.1. Glucose Biosensors

Diabetes is one of the foremost diseases due to which millions of people worldwide are suffering. According to recent statistics released by the International Diabetes Federation, 425 million adults globally are estimated to have diabetes, and this number could rise to 629 million by 2045 [86]. An acceptable range for blood glucose levels prior to meals is 70–99 mg/dL, provided that there is at least an 8 h interval between them. A glucose level of 100–125 mg/dL is indicative of pre-diabetes, and a level of 126 mg/dL or more is indicative of diabetic disease [87]. Anomalous fluctuations in blood glucose levels can result in major health problems, harming the kidneys, retina, brain, heart, leg, and other blood vessels, among other organs. As a result, accurate, timely blood sugar level monitoring and detection are essential components of a thorough clinical diagnosis. Hence, there is a greater need for reliable and steady gadgets that can sense or detect blood glucose levels. The first results on glucose concentration detection with an enzyme electrode derived from glucose oxide were published by Clark and Lyones et al. [88,89]. There are three different kinds of electrochemical glucose sensors. First, the electrode modified with glucose enzyme to facilitate detection. The reaction between the enzyme-modified electrode and glucose in the presence of O_2_ forms H_2_O_2_. The amount of glucose is then determined using the produced H_2_O_2_. The second kind of detection involves the use of electron mediators, which accelerate the kinetics of electron transfer between the glucose oxide active center and the electrode surface. Nevertheless, there are a number of issues with these mediators that could lower overall energy and lead to instability. Enzymatic and non-enzymatic biosensors witnessed swift advancements in the third category, facilitating direct reaction kinetics between the electrode surface and the active center of glucose oxide, obviating the necessity for intermediaries involved in electron transfer [90]. Despite significant advances in these biosensors, operational stability remains a major challenge. Therefore, to address the issues mentioned earlier and assess the optical and electronic kinetics of these systems, multiple kinds of macrocyclic compounds have been targeted as active catalysts on the working electrode. Chemically synthesized Pcs, polymers, macromolecular carbon nanostructures, and bimetallic macrocycles with various functional groups, such as carbonyls, thiols, amines, acidic hydroxyls, or -OH of acids, serve as a convenient matrix for immobilizing the electrode substrate [91]. Furthermore, macrocycle composite materials containing different types of conductive nanostructured carbon are used. A smart strategy for creating functional electrodes for biosensors is to incorporate macrocycles onto the surface of highly conductive carbonaceous materials. This integration increases surface area, electron conductivity, and a large number of active sites that provide a high surface for analyte molecules to interact. As an illustration, Qi et al. [92] attached a gold nanostructure to a graphene oxide (GO) nanosheet using benzene bridging and aryldiazonium salt reactions. On GO, phenyl bridged gold nanoparticles (AuNPs) were formed and bonded to 4-aminophenol modified GCE. The functionalization of 4-carboxyphenyl (CP) and glucose oxidase (GOx) through covalent attachment resulted in the GCE/GO-Ph-AuNPs-CP/GOx electrode, which is utilized directly for glucose sensing. The designed electrode exhibited a sensitivity of 42 mA.mM^−1^ and higher activity in the linear range of 0.3 to 20 nM. Due to their high specific surface area, excellent biocompatibility, and stability, the gold nanoparticles on GO-Ph-AuNP act as electrical nano-plugs, enabling interaction with the redox-active center of GOx. Despite their promising activity, the fabrication of such electrodes is complex due to the extensive experimental parameters and processes involved. To address this challenge, researchers have made considerable efforts in designing and developing glucose sensors with simplified fabrication processes, utilizing a range of straightforward hybrid composites comprising GO and highly redox-active materials. For instance, nonenzymatic nickel cobalt alloy nanoparticles (NiCoNps) are extensively used as active catalyst materials for the detection of glucose and hydrogen peroxide (H_2_O_2_) [93]. Similarly, nickel cobalt alloy nanoparticles anchored on polypyrrole/reduced graphene oxide nanocomposites, i.e., a NiCoNPs/polypyrrole/rGO nanocomposite-based glucose biosensor, was fabricated by Sheng et al. [94]. The improved electrocatalytic activity was progressively shown by the modified electrode with a sensitivity of 153.5 mA·mM^−1^·cm^−2^, linear range of 0.5 µM to 4.1 mM, and LOD of 0.17 µM. In a similar vein, Omotayo Adeniyi et al. [95] created an electrode for the detection of glucose in human saliva that was modified with a single-walled carbon nanotube/reduced graphene oxide/cobalt Pc nanohybrid on GCE (GCE-SWCNT/rGO/CoPc). The electrode has a linear range of 0.30 μM to 0.50 mM, sensitivity of 992.4 μA·mM^−1^·cm^−2^, and LOD of 0.12 μM. The superior catalytic activity of the electrode was achieved by the synergetic effects between Pcs molecules and CNTs, leading to an increase in surface area, conductivity, and binding capacity. However, this electrode material is limited for micromolar concentrations. Thus, in pursuit of lower detection limits and higher sensitivity in glucose sensing, Mounesh et al. [96] developed a glucose biosensor utilizing a hybrid composite of multiwalled carbon nanotubes (MWCNTs) and a macrocycle, i.e., aluminum (III) Pc macromolecule (AlPc/MWCNTs), which involves π-π interactions. For the purpose of detecting glucose, the designed hybrid composite was drop-casted onto GCE. The sensor successfully demonstrated a broad linear range of 50–500 µmol, a sensitivity of 0.058 µA·mM^−1^·cm^−2^, and a lower LOD of 2.5 nM. Additionally, the fabricated electrode was capable of sensing H_2_O_2_ in the nanomolar range. However, the electrode exhibited deviation in current response at higher concentrations of glucose, resulting in a higher LOD value (18 nm/L). Furthermore, the authors did not provide any insight into the deeper mechanism or the active sites responsible for sensing and signal amplification. To explore stable catalysts and their mechanism towards sensing of glucose biomolecule, Wang et al. [97] employed bimetallic metal–organic frameworks (MOFs) with Co and Ni as metallic sources because of their greater abundance, low-cost, and high electrocatalytic activity. Firstly, a range of bimetallic MOFs, Ni_2_Co1-L MOFs, with various ligands (L), such as H_2_BPDC (4,4-biphenyldicarboxylic acid), H_2_NDC (2,6-naphthalenedicarboxylic acid), and H_2_BDC (1,4-benzenedicarboxylic acid), were prepared via straightforward stirring method at room temperature. The effects of various metal centers, ratios, and ligand substitution on the electrocatalytic activity was investigated. Among the catalysts used, Ni_2_Co_1_-BDC (Nickel cobalt-benzenedicarboxylic acid) on GCE exhibited the highest electrocatalytic activity for glucose sensing due to its favorable disc-like structure, which provides more surface area in terms of active sites, and the mechanism of the redox process at the electrode is represented by Equations (1)–(4). The electrode has a linear range of 0.5–2899 µM, sensitivity of 3925 µA·mM^−1^·cm^−2^, and LOD of 0.29 µM. The higher catalytic activity demonstrated for glucose resulted from the hierarchical sheet-like Ni-BDC structural modification. This modification yields large accessible active sites, including an interconnected 3D structure that provides interspaces for the diffusion of biomolecules. This structure also reduces contact resistance and enhances the mass or charge transfer rate at the interface between the electrode and the electrolyte.

Ni^2+^-H_2_BDC → Ni^3+^-H_2_BDC + e^−^ (1)

Ni^3+^-H_2_BDC + OH^−^ + glucose → Ni^2+^-H_2_BDC-glucolactone + H_2_O + e^−^ (2)

Co^2+^-H_2_BDC → Co^3+^-H_2_BDC + e^−^ (3)

Co^3+^-H_2_BDC + OH^−^ + glucose → Co^2+^-H_2_BDC-glucolactone + H_2_O + e^−^ (4)

**Figure 1:**
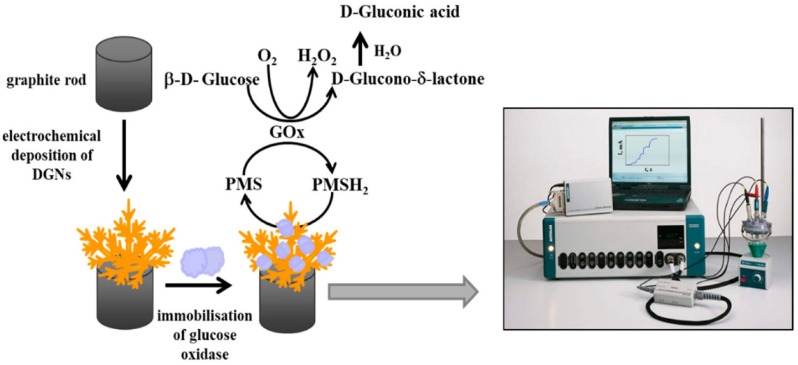
Schematic representation of electrochemical DGN deposition on graphite rod electrodes followed by glucose oxidase immobilization and glucose determination by the developed biosensor, (adopted from Ref. [98]).

Nowadays, gold micro- and nanostructure derivatives have garnered considerable attention owing to their size and shape-dependent physical and chemical properties. Optimized dendritic gold nanostructures (DGNs) modified on a graphite rod (GR) electrode, were used as active catalyst to demonstrate a stable glucose biosensor by Ramanaviciene et al. [98]. First, the DGNs were deposited on the graphite substrate using an electrochemical deposition technique that included DPV, constant potential, and pulse amperometry-without the stabilizers. Furthermore, the ideal parameters for improved deposition are an AuCl_4_ concentration of 6.0 mmol L^−1^, a DGN synthesis time of 400 s, and an electrodeposition potential of −0.4 V. Additionally, the FESEM (field emission scanning electron microscopy) method was employed to analyze the atom arrangement of the modified electrode. This analysis was crucial because the overall efficiency of the working electrode primarily depends on the morphology of the DGNs formation on the GR electrode surface. Consequently, the authors optimized the process to yield highly efficient and stable biosensor. As illustrated in Figure 1, the electrode was constructed by first adsorbing the enzyme glucose oxidase on the surface of DGNs, then creating a covalently cross-linked vapor with glutaraldehyde. The developed electrode effectively detects glucose over a wider range of up to 9.97 mmol/L, with a LOD of 0.059 mmol/L.

**Figure 2:**
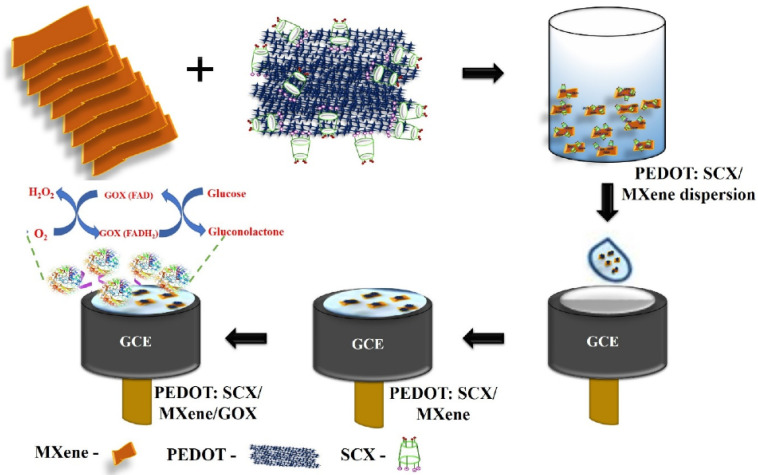
Schematic representation of electrode modification using glucose oxidase/PEDOT:4-Sulfocalix[4]arene/MXene composite for the glucose biosensor, (adopted from Ref. [99]).

In order to fabricate a simple electrode to exhibit lower LOD, Murugan et al. [99] immobilized GO_X_ onto poly(3,4-ethylenedioxythiophene):4-sulfocalix[4]arene (PEDOT:SCX)/MXene. This electrode was then employed to detect minimal glucose levels. Initially, 4-sulfocalix[4]arene was used to prepare PEDOT through an oxidative chemical method. Conversely, they created 2D-layered MXene in the presence of N_2_ atmosphere at higher temperatures. After that, these two compounds were divided in a 1:1 ratio to create a hybrid composite film, which was then ultrasonically processed. The film was then immobilized for electrochemical analysis on the modified GCE with chitosan, as shown in Figure 2. The produced electrode displayed a FAD-GO_X_ (flavin adenine dinucleotide glucose oxidase)-redox couple with a formal potential of −0.435 V, which is ascribed to direct electron transfer occurring between the electrode surface and the enzyme, thereby enhancing the electrocatalytic activity of glucose sensing. Even after 20 days, the hybrid composite film produced good reproducibility and successfully demonstrated a linear range of 0.5 to 8 mM and LOD of 22.5 µM. Table 1 presents an overview of the electrochemical activity of different hybrid composites based on macrocycle-carbon nanoparticles with respect to glucose sensing, including sensitivity, selectivity, linear detection range, and LOD [89,90,91,92,93,94,95,96,97,98,99,100,101,102,103,104,105,106,107,108,109,110,111,112].

### 4.2. Hydrogen Peroxide (H_2_O_2_) Biosensors 

As discussed earlier, glucose serves as a primary energy source for cells. It undergoes glycolysis to produce pyruvate, which then enters the citric acid cycle (Krebs cycle) and oxidative phosphorylation, and ultimately generates adenosine triphosphate (ATP) for cellular energy. Throughout these metabolic processes, reactive oxygen species (ROS) such as H_2_O_2_ are produced as byproducts through various pathways, including the electron transport chain in mitochondria. ROS play a dual role in cellular physiology: they act as signaling molecules in low concentrations but may be harmful when they are in excess, leading to oxidative stress and damage to cellular components such as proteins, lipids, and DNA. In this regard, researchers have been paying close attention to H_2_O_2_ because of its powerful oxidizing and reducing abilities, which has led to a resurgence of its use in the pharmaceutical, food, medical, and environmental monitoring industries [113,114]. Furthermore, H_2_O_2_ traverses cell membranes in a variety of biological tissue chambers and is thought to be a highly reactive oxygen species involved in a variety of biological processes. Additionally, it is produced as a byproduct of oxidation-related enzymatic reactions and is essential for altering biological signals. Owing to the human body’s uneven H_2_O_2_ concentrations, it has been found to be a significant biomarker in living cells, especially for the detection of cancer. In addition, it plays a crucial physiological role in conditions like Alzheimer’s, myocardial infarction (heart attack), and atherosclerosis [115]. Owing to the detrimental impacts of H_2_O_2_ on the human body and biological system, its detection is desperately needed. H_2_O_2_ can be detected using a wide range of methods, such as chromatography [116], titrimetry [117], chemiluminescence [118], spectrophotometry [119], fluorescence [120], and phosphorescence [121] with a lower LOD of roughly 10^−11^ M. However, there are drawbacks associated with traditional methods, like complexity, cost, time, and the need for a lot of reagents. On the other hand, due to their low limit of quantification (LOQ), low LOD, easy fabrication, fast response, high sensitivity, and selectivity, electrochemical methods have become the preferred techniques for H_2_O_2_ detection [122,123]. As such, the development of a dependable H_2_O_2_ sensor addresses the emphasis related to precision, sensitivity, effectiveness, and quick detection. To overcome these issues, chemical reactions with H_2_O_2_ are made possible by the interconnection of electrocatalysts on the surface, which can produce a variety of products depending on the applied potential. Thus far, the electrochemical method utilizing different nanomaterials-based metal electrocatalysts has allowed researchers to successfully detect H_2_O_2_ in micro- to nanomolar concentrations. However, issues like limited availability, poor stability, greater expenses, and insignificant anti-interference continue to be the major obstacles to the widespread applications of metal electrocatalysts based on nanomaterials. The various structural characteristics of organic complexes based on macrocycles and their hybrid composites have been thoroughly investigated for their potential as stable electrocatalysts or as functional electrodes in the development of H_2_O_2_ biosensors. These hybrid catalysts overcome challenges such as higher surface area [124], chemical [125], thermal [126,127], and electrochemical stability [128], biocompatibility [129], increased electrical conductivity [130,131], and dynamic electrocatalytic stability [132].

The H_2_O_2_ sensing activity is blocked by conventional electrodes, which have a higher reduction overpotential. By adding macrocycle-based redox-active catalysts to pre-cleaned working electrodes, many attempts have been made to improve electron transfer kinetics and lower the overpotential. Like peroxidase, H_2_O_2_ sensing is electrocatalytically active and is dependent on a number of physical parameters, including pH, temperature, and so on. The ideal temperature range for catalytic activity is between 35 and 40 °C. The mechanism for the electrocatalytic detection of H_2_O_2_ in phosphate buffer solution (PBS) on an active surface of the modified electrode is as follows:

(5) $$H_{2}O_{2}+e^{-}↔{{(H}_{2}O}_{2}^{-}{)}_{\mathrm{ads}}$$

(6) $${{(H}_{2}O}_{2}^{-}{)}_{\mathrm{ads}}+e^{-}↔(2HO^{-}{)}_{\mathrm{ads}}$$

(7) $$(2HO^{-}{)}_{\mathrm{ads}}+{2H}^{+}↔2H_{2}O$$

The first step (Equation (5)) is the adsorption of reducible H_2_O_2_ on the surface of the electrode, and the kinetics of this process are directly correlated with the electrode surface’s active sites. In the electrochemical reduction process, the reductive dissociation of adsorbed H_2_O_2_ via Equation (6), requires the cleavage of the O-O bond to overcome the high-energy barrier, and is the rate-determining step. This step is totally dependent on the amount of H_2_O_2_ that has been adsorbed on the electrode’s active surface. The linked OH^−^ groups are protonated in the last step (Equation (7)), resulting in the formation of water molecules that desorb from the electrode surface. The strong oxidation property of the first step’s product (H_2_O_2_^−^) may cause damage to the electrode as it is adsorbed on the catalytic surface [133]. The development of an electrocatalyst with an increased catalytic activity is required to address this problem by improving electron transfer kinetics and reducing the generation of intermediate products. Due to their high porosity, thermal stability, large surface area that can accommodate active sites, and combination of inorganic and organic properties, hybrid materials based on macrocyclic systems are being considered as a potential solution to this problem [134,135]. Their hybrid composites are more densely packed, have evenly spaced active sites, and have porosity that encourages the electrocatalyst’s and substrate’s active sites to interact [136]. These parameters significantly improve signal amplification capability, stability, and sensitivity. Furthermore, charge transfer kinetics are aided by the C-O-M (carbon–oxygen–metal) bonds found in porphyrins [137], Pcs [138], and metal–organic frameworks [139] which function as electron transport tunnels. An H_2_O_2_ biosensor was made by M.-Y. Hua et al. [140] using dopant-type polyaniline-polyacrylic acid composite (PAn-PAA) films on a gold (Au) substrate. With a broad linear range between 0.04 and 12 mM and a lower LOD value of 0.02 mM, the designed electrode demonstrated a sensitivity of 417.5 μA·mM·cm^−2^. In a neutral environment, the hybrid composite electrode displayed increased activity.

PAA + H_2_O_2_ → peroxy-acid (8)

PAn + peroxy-acid → PAn N-oxide(9)

PAn N-oxide + 2e^−^ + 2H^+^ → PAn + H_2_O (10)

**Figure 3:**
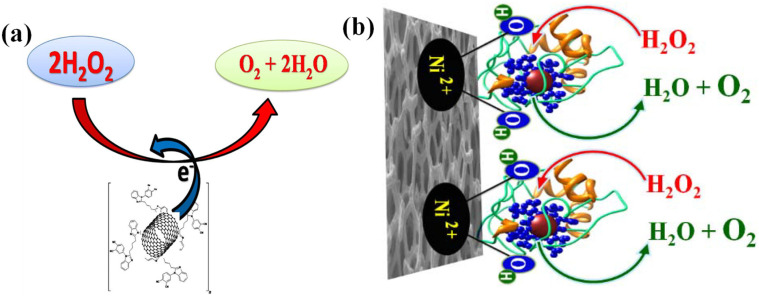
(**a**) Schematic diagram of electrochemical reduction of H_2_O_2_ on Poly(CoTBImPc/GCE electrode (adopted from Ref. [141]) and (**b**) electro-catalytic oxidation of H_2_O_2_ over Ni foam decorated by Hb (adopted from Ref. [143]).

Three steps were involved in the detection of H_2_O_2_. In order to create peroxy-acid, the added H_2_O_2_ first endured a process of oxidation with the carboxyl group of PAA (Equation (8)). Subsequently, this peroxy-acid chemically oxidized the aniline’s amine group to produce imine-N-Oxide (Equation (10)). Ultimately, the attached imine-N-oxide reversed back to PAn and water through electrochemical reduction, which involved the gain of electrons (Equation (10)). The hybrid PAn-PAA composite electrode exhibited increased stability due to the inherent properties of PAn. By combining PAA and PAn, a high porosity structure was produced, greatly increasing the composite electrode’s surface area for H_2_O_2_ reaction. Using a straightforward technique, Lokesh et al. worked on the electrochemical sensing of H_2_O_2_ using various hybrid composite catalysts made of cobalt phthalocyanine macrocycles. They designed an organic N4-macrocyclic polymeric cobalt tetrabenzimidazole phthalocyanine known as (Poly(CoTBImPc)) [141] and utilized CV technique to verify the redox character of the synthesized Poly(CoTBImPc). The electrode exhibited improvement in sensitivity, conductivity, and surface area after being physically mixed with carbon nanotubes (CNTs) and Poly(CoTBImPc). As shown in Figure 3a, the composite electrocatalyst was drop-casted on the GCE to detect H_2_O_2_ electrochemically. After being examined using CV and amperometric methods, the developed electrode showed improved stability and repeatability without sacrificing its characteristics. It also showed a wide linear range of 10 to 100 nM H_2_O_2_ concentration, with a nanomolar LOD of 2 nM. The composite electrode’s activity is primarily ascribed to the polymeric Pc and CNTs’ synergistic effects, which increase the number of active sites available for H_2_O_2_ sensing. In a similar way, they also designed electropolymerized film of cobalt tetrabenzimidazole phthalocyanine, poly(CoTBIPc) [142]. This poly(CoTBIPc) was then deposited on GO, which exhibited better sensing property towards reduction of H_2_O_2_. The fabricated GCE/GO/Poly CoTBIPc electrode showed a detection linear range from 2 to 200 µM with a better LOD of 0.6 µM. Furthermore, this electrode exhibited greater reproducibility, stability, and high selectivity towards reduction of H_2_O_2_ even in the presence of several interference compounds. However, the exact active sites for the H_2_O_2_ sensing and its suitable mechanism are not provided. In order to gain deeper understanding of peroxide detection, Akhtar et al. [143] presented a simple and highly sensitive non-enzymatic H_2_O_2_ biosensor using a composite made of functionalized hemoglobin (Hb) macromolecules on 3D-structured Ni foam substrates, as demonstrated in Figure 3b. Even after immobilization, the Hb on the electrode continues to function biologically through its electrical network. The combination of the facile electron transfer kinetics and bioactive properties of Hb, along with the remarkable physical and inherent catalytic activities of 3D-structured Ni foam, demonstrated a rapid response for H_2_O_2_ sensing with a broad linear range of 50 μM to 850 μM, LOD of 0.41 μM, and a higher sensitivity of 0.39 μA·mM^−1^. The designed non-enzymatic Hb-modified Ni foam demonstrated a higher response. This work demonstrates that the Hb-coated Ni foam exhibits significantly improved electrocatalytic activity for H_2_O_2_, characterized by enhanced reversibility, fast electron charge transfer kinetics, and a higher current response compared to bare Ni foam electrodes. The enhanced activity can be attributed to two key factors:

(i) The bulkier structure of Hb contributes to the activation of the electrode surface and facilitates electron transfer kinetics without impeding the diffusion of hydroxide ions through the 3D porous network. Surface modification of Hb targets the heme cofactor, which consists of a ring of conjugated double bonds surrounding an iron atom. These iron atoms possess narrowly spaced energy levels, allowing for easy electron transfer facilitated by extra conjugation of double bonds. This phenomenon prevents energy loss as heat and enables energy conversion into smaller processes, such as proton pumping across a membrane or metal reduction [144].
(ii) The 3D structure of Hb features a configuration where hydrophobic amino acid clusters are buried inside the molecule, while hydrophilic residues are located towards the surface [145]. This structural arrangement enhances the hydrophilicity of the electrode surface and increases the number of active sites compared to bare Ni foam electrodes, as illustrated in Figure 3b.

**Figure 4:**
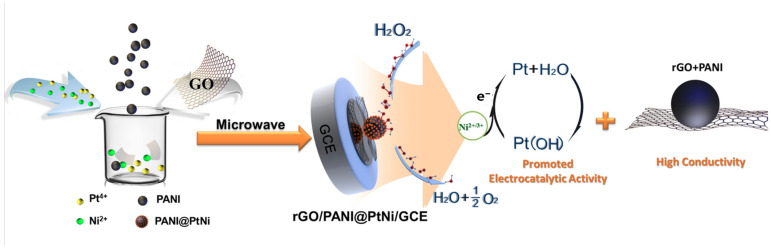
Scheme for fabricating the modified glassy carbon electrode (GCE) with platinum–nickel decorated polyaniline nano-sphere (rGO/PANI@PtNi) nanocomposite for H_2_O_2_ sensing, (adopted from Ref. [146]).

For nonenzymatic H_2_O_2_ sensing, Bing Du et al. [146] designed a hybrid composite material decorated with polyaniline nanospheres on reduced graphene oxide (rGO/PANI@PtNi) using microwave technique, as depicted in Figure 4. In comparison to pure rGO, the designed composite material (rGO/PANI@PtNi) has a larger surface area because of the attachment of highly conductive PANI nanospheres, which facilitate better dispersion of PtNi nanoparticles rather than agglomeration. Furthermore, the distinct oxidation states of Ni^2+^/^3+^ in the PtNi alloy sufficiently enhance Pt electrocatalytic activity. The synergistic interaction between these materials with improved electrical conductivity results in increased electrocatalytic activity. With a linear range of 0.1–126.4 mM and a LOD of 0.5 µM, the rGO/PANI@PtNi hybrid composite on the GCE exhibited excellent sensitivity. It also showed better stability for over a week without any degradation. The reaction mechanism for H_2_O_2_ sensing at the rGO/PANI@PtNi hybrid composite primarily relies on the facilitation of Ni hydroxides for the electrocatalytic activity of Pt. Initially, the dissociation of H_2_O_2_ occurs on the Pt surface, where it is transformed into -OH groups by occupying available sites on the Pt surface. Subsequently, the adsorbed -OH on Pt undergoes electrochemical reduction, forming water with the evolution of oxygen gas, as depicted in Figure 4. This regeneration process enables the active Pt sites to regenerate, making them available for further dissociation of H_2_O_2_. The electrochemical activity of several hybrid composites for H_2_O_2_ detection is compiled in Table 2 with respect to sensitivity, selectivity, linear detection range, and LOD [140,141,142,143,144,145,146,147,148,149,150,151,152,153,154,155,156,157,158,159,160,161,162,163].

### 4.3. Uric Acid (UA) Biosensors

**Figure 5:**
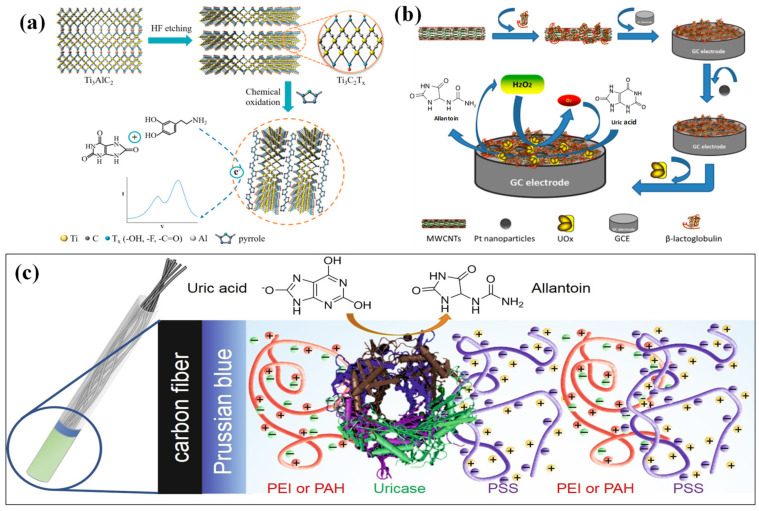
(**a**) Schematic illustration of the synthesis of the MXene/PPy nanocomposite (adopted from Ref. [164]), (**b**) schematic diagram of the reaction principle and preparation process of BSA/BLG-MWCNTs-PtNPs/GCE for uric acid sensing (adopted from Ref. [165]), and (**c**) immobilization of UOx onto carbon fiber electrode with PB redox mediator coated by PEI(PAH)/PSS layers (adopted from Ref. [166]).

In the human body, uric acid (UA) is the metabolite of purine [164] and is closely related to formation of kidney stone, diabetes, and heart diseases [165]. UA, also known as 2,6,8-trihydroxypurine, is a crucial purine metabolic intermediate [166]. Typically, the range of stable concentrations for UA is maintained, and the amount of UA generated by the human body during normal activities is nearly identical to its metabolic parole [167]. Urine, blood, serum, and other body fluids contain the vital biomolecule UA, whose concentration variation leads to different abnormalities and diseases. In a healthy individual, the normal range for uric acid concentration is 1.2 × 10^−4^ to 4.5 × 10^−4^ mol·L^−1^ in blood/serum and 2 × 10^−3^ mol·L^−1^ in urine [164,168]. However, the concentration of UA excretion in urine is 25–74 mg·dL^−1^, and this concentration is primarily influenced by purine metabolism. Unequal variation in the amount of UA in human body fluids (blood or urine) may serve as an indicator for a number of diseases, including hyperuricemia, kidney damage, Lesch–Nyhan syndrome, gout, pregnancy toxemia, high blood pressure, and cholesterol. Additionally, an increase in the UA concentration in blood level can lead to cardiovascular disease. Oxidative stress and multiple sclerosis disorders appear to be associated with UA concentration as well [169,170,171]. As a result, a critical component for medical diagnosis is the tracking and sensing of UA concentration levels in the human body. Several techniques are employed by researchers from across the globe to identify UA, such as direct electrochemical sensing, chemiluminescence, single line manifold, uricase immobilization techniques, HPLC (high-performance liquid chromatography), and fluorescence. With advancements in quick response [172,173], ease of operation [174,175], specificity [176,177], low cost [178], and high sensitivity [179], electrochemical sensing is one of the most widely used and highly regarded techniques for measuring low levels of UA. In order to enhance catalytic features, Dong et al. [164] synthesized a two-dimensional MXene-type titanium aluminum carbide (Ti_3_C_2_T_x_). As shown in Figure 5a, this composite material is immobilized on GCE in order to study the electrochemical determination of UA. Initially, the etching technique was used to prepare the multilayers of Ti_3_C_2_T_x_-MXene, which then delaminated in aqueous hydrofluoric acid. Subsequently, PPy nanostructures with a diameter of approximately 200 nm were composited with an MXene layer using a standard oxidation process. A major obstacle to more useful applications is the controlled preparation method and PPy material signal amplification. In order to balance magnitude and density during the synthesis of PPy nanostructures to increase the conductivity as well as the electrochemical active surface, a general chemical oxidative method was employed with modification. With a broad linear range of 50–500 μM and a LOD of 0.15 μM of UA, the designed MXene/PPy on GCE successfully oxidizes the added UA even in the presence of ascorbic acid (AA). The preparation of this electrode involved complexity with a multistep procedure and, in addition, the fabricated electrode exhibited poor sensitivity towards the targeted molecule as compared to other electrodes.

Chen, Q. et al. [165] used a simple and efficient electrochemical biosensor for UA. In order to achieve better dispersion, the authors prepared the hybrid material by dispersing functionalized MWCNTS in β-lactoglobulin (BLG) solution for 60 min. BLG-MWCNTs were then extracted and centrifuged. Using an adsorption technique, the prepared BLG-MWCNTs composite was coated on GCE that was previously cleaned. Subsequently, BLG-MWCNT-modified GCE was electrodeposited with platinum nanoparticles (PtNPs) with the following parameters: 10 mM K_2_PtCl_6_, 0.1 M H_2_SO_4_, −750 mV, 400 s. This resulted in a hybrid composite electrode consisting of BLG-MWCNTs-PtNPs/GCE. Ultimately, the bull serum albumin (BSA) was used to covalently bond the urate oxidase (UOx) on BLG-MWCNTs-PtNPs/GCE. This combination of BSA/BLG-MWCNTs-PtNPs/GCE is used for the electrochemical measurement of UA, as illustrated in Figure 5b. In this case, the UOx oxidizes the added UA to produce allantoin and H_2_O_2_. With a linear range of UA detection of 0.02 to 0.5 mM, sensitivity of 31.131 μA mM^−1^, and a LOD of 0.8 μM, the authors were able to obtain a better response for UA detection. However, the primary drawback of this electrode was its limited capability to exhibit enhanced activity only within an electrolyte solution of below pH 7, and reduced activity was observed in solutions with pH greater than 7. Additionally, the authors did not provide an explanation regarding the exact factor or active site responsible for UA detection in this context.

An advanced polyelectrolyte crowding concept was proposed by Nikolaev, K.G. et al. [166] for the creation of an enzyme-based biosensor to measure the concentration of UA. This technique has also been used as a prognostic indicator and a tool for tracking the disease in COVID-19 patients. The conductive electrode substrate utilized was carbon fiber that had been modified with a hybrid composite of a layer of urea oxidase (UOx), a redox mediator called Prussian blue (PB), and a layer of polyelectrolyte that had been assembled layer by layer to form a complex coacervate composed of two types of polyelectrolytes: one that was highly charged poly(sodium 4-styrenesulfonate) (PSS) and the other which was weakly charged polyethyleneimine (PEI), poly(allylamine hydrochloride) (PAH) and their charges (+/−) are mentioned by colouring. The process of depositing film is quite difficult and is regulated by a number of methods, including quartz crystal microbalance (QCM) technique, which shows uniform distribution of polyelectrolyte layers on carbon fiber, cyclic voltammetry, and scanning electron microscopy (SEM) in conjunction with energy-dispersive X-ray (EDX) analysis (during the phase of PB deposition), and the schematics of this process is displayed in Figure 5c. The designed electrode exhibits a linear range of 10^−4^–10^−6^ M, a linearity dependence of 0.81 μM, and a higher sensitivity of 10.64 mA/μΜ for uric acid detection. By increasing the number of polyelectrolyte layers, the fabricated electrode successfully demonstrated higher reproducibility of UA levels in real urine samples of SARS-CoV-2 patients. Unfortunately, the sensor electroactivity data for the electrodes modified with polyelectrolyte multilayers, alternating with uricase layers, were insufficient for a direct comparison with the sensor in this study. However, the authors claimed that this designed sensor offers a lower usage of uricase enzyme and a less costly substrate (carbon fiber electrode vs. platinum). Thus, the carbon fiber-based layer-by-layer assembled uricase biosensor holds promise for clinical use due to its affordability, portability, and relatively straightforward design. The measured parameters by different hybrid composite modified electrodes towards UA sensing are listed in Table 3 [164,165,166,167,168,169,170,171,172,173,174,175,176].

### 4.4. Ascorbic Acid (AA) Biosensors

Numerous plants contain ascorbic acid (AA), commonly referred to as vitamin C [180], which is an important biomolecule and an antioxidant [181]. Because it plays a major role in numerous bodily metabolic processes, AA has been used widely as an oxidant in food and soft drinks, as well as in pharmaceutical applications [182]. Due to its potent oxidant capacity, it supports wound healing, iron absorption from non-heme systems, osteogenesis, collagen formation, and preservation of capillaries, bones, and teeth. It is also easily soluble in water and greatly aids in triggering an immune response [183]. Physicians strongly recommend it for the treatment of multiple illnesses, including Alzheimer’s, infertility, HIV infections, cancer, and atherosclerosis [184]. Generally, healthy human plasma has an AA concentration level between 50 and 70 µM [185]. Increased AA concentrations can cause gastric irritation and renal problems, while AA deficiency causes scurvy [186]. In view of the above concerns, it is extremely important to determine and record the AA concentration in the agricultural sector. To determine the amount of AA, a variety of methodologies have been used, such as iodometric, fluorometric, enzymatic, and electrochemical (EC) methods. Among these, EC methods have sparked curiosity due to their ease of use, pace, and affordability in measuring AA via electrochemical oxidation. Despite being a major oxidant, AA is difficult to detect electrochemically by direct oxidation because it interferes with other biomolecules that have similar oxidation potentials, such as UA and dopamine. Thus, the electroanalytical field is paying close attention to the development of convectional electrodes for AA measurement in the presence of numerous interfering species.

**Figure 6:**
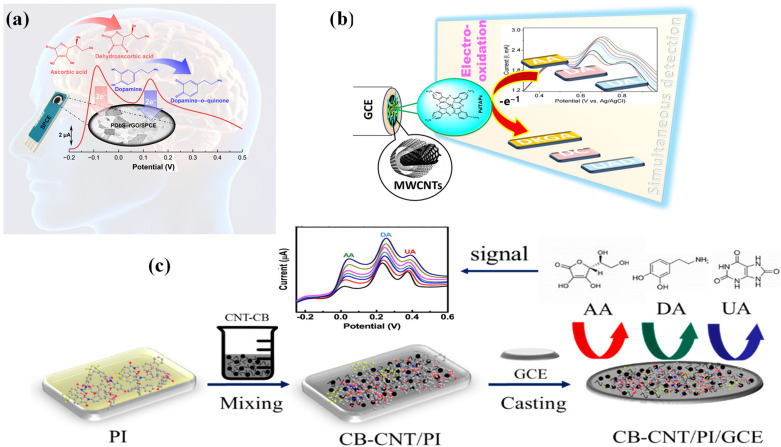
Schematic of (**a**) proposed PD*b*S–rGO/SPCE sensor for the simultaneous determination of AA and DA (adopted from Ref. [180]), (**b**) fabrication of PdTAPc/MWCNTs/GCE electrode for biomolecule sensing (adopted from Ref. [184]), and (**c**) fabrication of carbon black–carbon nanotube (CB-CNT)/polyimide (PI)-modified GCE for the detection of ascorbic acid (AA), dopamine (DA), and uric acid (UA) (adopted from Ref. [185]).

Chang, S.-C et al. [180] proposed an electrochemical biosensor that can detect ascorbic acid (AA) and dopamine (DA) by coating a hybrid composite made by incorporating rGO with a newly synthesized block copolymer, poly(DMAEMA-b-Styrene) (PDbS), on a screen-printed carbon electrode (SPCE). Various spectro-analytical techniques were used to characterize the fabricated poly(DMAEMA-b-Styrene) immobilized rGO on SPCE(PDbS–rGO/SPCE) electrode. With regard to DA and AA analytes, the hybrid composite electrode (PDbS–rGO/SPCE) demonstrated better peak to peak estrangement and sensitivity. The dynamic linear range of 10–1100 µM and LOD of 0.88 µM are displayed by the PDbS–rGO/SPCE electrode. Furthermore, even after being stored for four weeks, the designed electrode still demonstrated improved reproducibility and intrinsic interference capability. In order to illustrate the usefulness of this electrode design for measuring AA concentration, the authors also assessed the ex vivo brain tissue from a mouse model of Parkinson’s disease as indicated in Figure 6a.

**Scheme 3:**
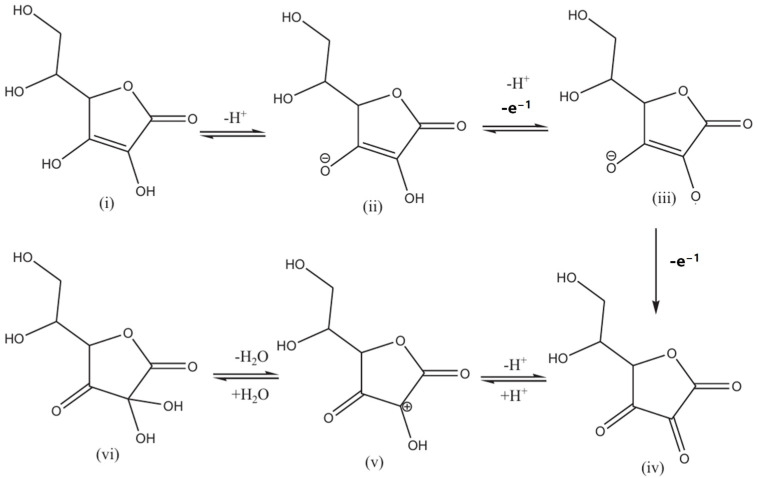
Proposed mechanism for the electrochemical oxidation of ascorbic acid.

However, a major draw-back of PD*b*S–rGO/SPCE electrode is its inability to function effectively at higher pH levels, specifically above pH 7. In order to detect three crucial biomolecules, i.e., DA, UA, and AA, M. Nemakal et al. [184] constructed a potent hybrid composite based on N4 macrocycle, palladium (II) tetraaminophthalocyanine (PdTAPc), with MWCNTs through physical mixing. The fabrication process of PdTAPc/MWCNTs on the GCE electrode as well as the oxidation mechanism on the electrode surface are depicted schematically in Figure 6b. The self-assembled monolayer (SAM) formation by the chemisorption method was as follows: Initially, authors immobilized PdTAPc on the electrode’s active surface area. The modified PdTAPc electrode was then decorated with MWCNTs to improve its conductivity and electron charge transfer. AA detection with the MWCNT-diffused PdTAPc-coated on GCE electrode is characterized by a linear range of 3–24 μmol·L^−1^, a LOD of 1 μmol·L^−1^, and a sensitivity of 1.0149 mA·μmol^−1^·cm^−2^. The developed electrode was also used to analyze a real sample of human urine. Further active sites for sensing applications are provided by the synergistic effects and π-π interaction between Pc molecules and MWCNTs. Additionally, the authors described the process by which AA is electrochemically oxidized on a developed electrode, as depicted in Scheme 3. The anionic product (ii) is produced in step (i) by the deprotonation of AA. Following the removal of one electron, compound (ii) is oxidized to form compound (iii). Dehydroascorbic acid (iv) (DHAA) is produced when compound (iii) undergoes faster electron transfer by releasing one electron. Since DHAA is an electroactive species, protonation produces compound (v). Compound (v) dehydrates to produce compound (vi), which is 2,3-diketogluconic acid (DKGA).

As shown in Figure 6c, Tao, D. et al. [185] established a novel composite electrode called the CB-CNT/PI/GCE electrode, i.e., carbon black (CB) and CNTs co-doped with polyimide (PI) on pre-cleaned GCE. This electrode was used for the sensing of AA, DA, and UA. The CB-CNT/PI/GCE demonstrated higher catalytic activity and, with the ternary mixture, the CV and DPV methods offer more accurate AA, DA, and UA detection. A linear range of 100–5000 µM, a LOD of 75 µM, and improved sensitivity are offered by the CB-CNT/PI/GCE hybrid composite electrode. For AA biosensors, the different hybrid composite electrocatalysts are compiled in Table 4 [180,181,182,183,184,185,186,187,188].

### 4.5. Dopamine Biosensors

**Figure 7:**
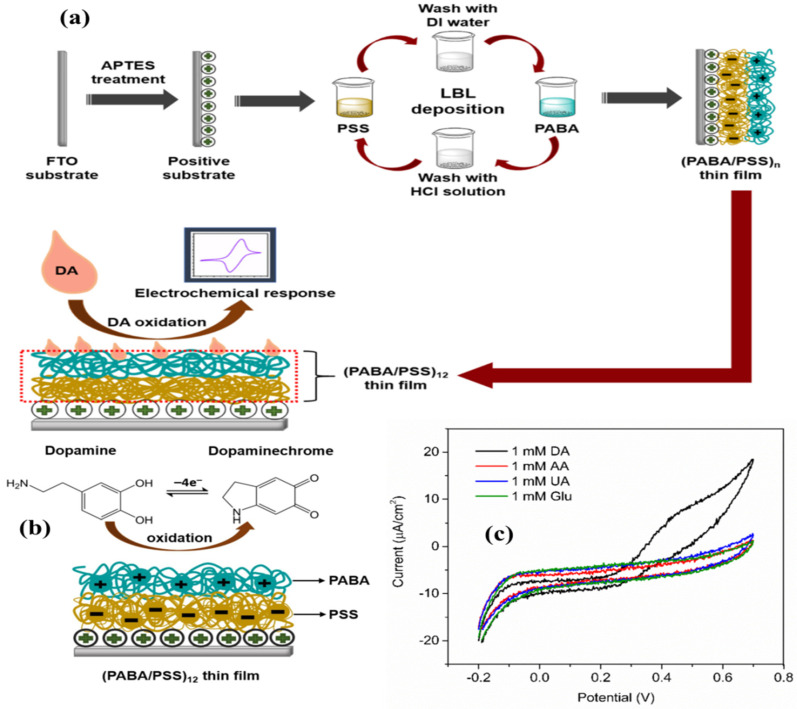
(**a**) Schematic presentation of PABA/PSS layer-by-layer film assembled for the electrochemical detection of DA on FTO substrate, (**b**) oxidation of dopamine to dopaminechrome, and (**c**) CV response of 12-bilayer PABA/PSS electrode for DA, AA, UA, and glucose at 100 mV·s^−1^ scan rate (adopted from Ref. [198]).

The seminal discovery of dopamine (DA) detection in 1958 by Carlsson et al. at the National Heart Institute of Sweden revolutionized our understanding of neurotransmitters [189], particularly its role in neurological disorders such as Parkinson’s disease [190]. Following this landmark discovery, electrochemical biosensors have emerged as crucial tools for DA quantification due to their real-time capabilities in monitoring the intricate oxidation and reduction processes of dopamine within biosensor platforms. Nevertheless, the accurate measurement of DA faces challenges due to the presence of electroactive interferents, including UA and AA, in biological samples [191]. In response to these challenges, innovative strategies have been developed to enhance analytical precision. The differentiation of DA levels between healthy individuals and Parkinson’s patients emphasizes the need for selectivity, especially in complex matrices like human serum [192]. This necessitates precise sample preparation, involving centrifugation and dilution for blood samples as well as urine samples [193]. Additionally, selective modification materials can be employed for miniaturized microelectrodes to analyze minute quantities in cerebrally extracellular fluid [194]. DA’s electroactivity involves oxidation and reduction within an electrochemical biosensor. At a precisely chosen potential, dopamine donates electrons to the electrode surface, generating a current proportional to its concentration. While this direct electroanalytical approach is rapid and sensitive, interference from other electroactive biomolecules stems from the use of enzyme-modified electrodes [195]. Tyrosinase, acting as the conductor of DA metabolism, catalyzes the oxidation of DA to DA o-quinone, amplifying the electrochemical signal while rejecting unwanted interferents. Alternatively, aptamers, acting as synthetic antibodies for DA, can be immobilized on the electrode, creating a lock-and-key recognition system [196]. Regardless of the chosen performance approach, the final step involves regeneration of the DA molecule to ensure the biosensor’s readiness for subsequent neurochemical interactions. Recent studies, such as the use of the calix(4)resorcinarene receptor in lipid membranes, have demonstrated quick DA detection with promising results. This intricate interplay of electrochemistry and biorecognition holds significant promise for transformative insights into neurological function and diseases [197]. The miniaturization and integration of electrochemical biosensors with microfluids and implantable platforms are crucial for real-time monitoring in complex biological environments. Recently, Panapimonlawat et al. utilized the layer-by-layer assembled film of poly(3-aminobenzylamine)/poly(sodium 4-styrenesulfonate)(PABA/PSS) as a dopamine biosensor on a fluorine-doped tin oxide (FTO) substrate. The optimized film has ability to detect DA in neutral solution. The 12-bilayer PABA/PSS electrode showed good DA sensing with a sensitivity of 6.922 nA·cm^−2^·µM^−1^, linear range of 0.1–1.0 µM, and LOD of 0.0628 µM. In addition, PABA/PSS thin film also exhibited good selectivity towards detection of DA even under the common interferences like AA, UA, and glucose, as presented in Figure 7 [198].

**Figure 8:**
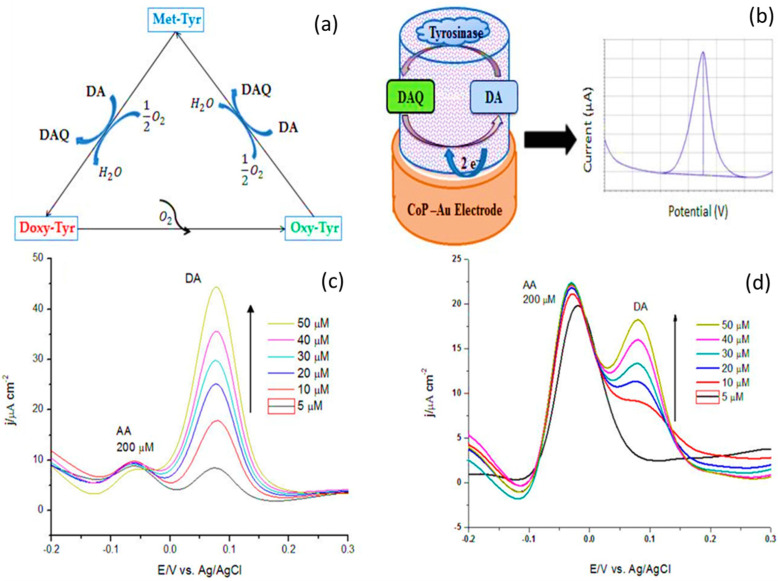
(**a**) Application of tyrosinase during catalysis of dopamine; (**b**) detection of DA at the CoP-Tyr surface; differential pulse voltammograms of (**c**) Au-CoP-Tyr biosensor and (**d**) Au-CoP-biosensor at 5–50 µM DA (adopted from Ref. [205]).

Recent advancements in biosensing materials, particularly macrocyclic compounds, have addressed challenges associated with miniaturization. Moreover, the choice of electrode materials significantly influences biosensor performance. While traditional metals and metal oxides like Pt and Au offer high conductivity and electrocatalytic activity for DA oxidation, the exploration of alternative materials, such as carbon nanomaterials (e.g., graphene and carbon nanotubes), transition metal dichalcogenides, and conducting polymers, is imperative due to their tunable properties and potential for miniaturization [199]. Recent studies by Keshavananda Prabhu et al. [200] on a macrocyclic-based iron–Pc complex with carbon nanoparticle-doped counterpart showcased exceptional performance in terms of linear range and LOD for DA oxidation. Similarly, Nobuhle et al. [201] reported efficient electrocatalytic catechol oxidation using CoPc–carbon nanomaterial conjugates. These conjugates, particularly involving single-walled carbon nanotubes, exhibited significant improvements in detection limits, sensitivity, and catalytic rate, highlighting their potential in biosensing applications. Mounesh et al. designed MWCNT substituted tetra-1-benzyl-1*H*-pyrazol-3-carboxamide cobalt(II) phthalocyanine using drop casting method [202]. The experiment was recorded within linear range 50 to 750 nmol L^−1^ with detection limits of 19 nmol L^−1^. Additionally, carbon-based materials, such as carbon nanotubes (CNTs), mesoporous, graphene, and nanocarbons, etc., exhibited sustain electrocatalytic activity and stability due to their large specific surface area, high electrical conductivity, and tunable physicochemical properties in electrochemical applications [203]. Despite these advantages, carbon-based materials majorly suffer from aggregation issues, poor dispersibility, limited pore size, surface defects, biocompatibility concerns, and high production costs [202]. In addition, a major challenge in DA biosensors remains the interference from electroactive species like AA and UA. Addressing this challenge requires exploring enzyme-based biosensors utilizing the selective catalytic activity of DA oxidase or tyrosinase for enhanced specificity [204]. Additionally, the development of molecularly imprinted polymers and aptamers tailored for DA holds promise for improved selectivity. Furthermore, advanced signal processing techniques and machine learning algorithms could be employed to discriminate against interferents and refine biosensor outputs. Figure 8a–d explain the utilization of tyrosinase-cobalt (II)-porphyrin (CoP)-fabricated biosensor for the electrochemical detection of DA. The reduction current of DA-quinone showed promise to measure the function of DA concentration with higher selectivity towards DA in linear range of 2–30 µM. Furthermore, the biosensor exhibited a sensitivity of 1.22 ± 0.02 µA·cm^−2^·µM^−1^ and a LOD 0.43 µM, with high reproducibility of 96% [205]. However, limited linear range, anti-interference for other interfering species, enzyme stability and activity, and long-term in vivo performance remain challenges.

**Figure 9:**
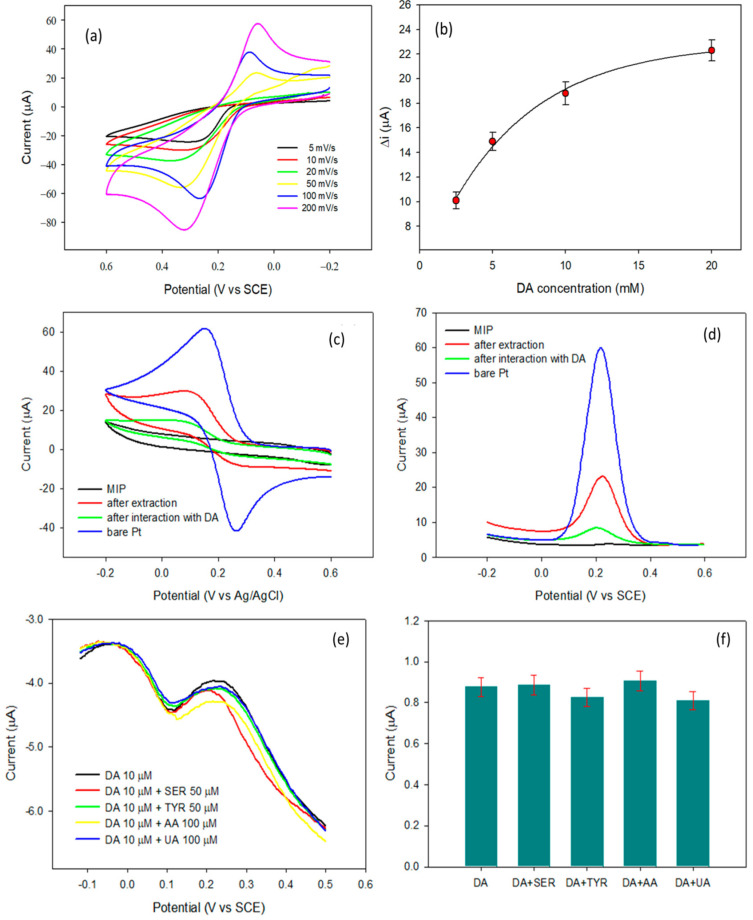
(**a**) CV for bare platinum electrode at different scan rates, (**b**) DA concentration in presence of molecularly imprinted polymers in the potential range of −0.1 to 0.9 V vs. SCE for 30 cycles, (**c**) CV profile of 5 mM of ferricyanide on a bare Pt electrode, (**d**) DPV for 5 mM of ferricyanide acquired in the same conditions of CV at a potential range between +0.6 V to −0.2 V with 4 mV potential increased for each time with amplitude of 50 mV, and pulse width 50 ms, pulse period of 0.2 s, and (**e**,**f**) effect of serotonin, tyramine, AA, and UA on the DPVs of 10 μM DA using a MIP-modified electrode (Adopted from Ref. [206]).

In addition, a preliminary investigation of DA’s electrochemical behavior on Pt, coupled with a meticulous evaluation of molecularly imprinted polymer (MIP) preparation conditions, proved crucial to overcome existing limitations. Notably, pH acts as a key factor to prevent unwanted DA polymerization at higher concentrations. This phenomenon allowed for successful deposition of poly(o-aminophenol) without DA involvement. Consequently, template molecules were easily extracted using an acidic solution, with high reproducibility and selective imprinted cavities within the MIP. The sensor exhibited high selectivity, with no interference observed, as presented in Figure 9a–f. The utilization of a MIP sensor was further validated by analyzing human urine samples, which showed good recovery percentage ranges from 92.5% to 109.1%, while the LOD may require further optimization [206]. This overall performance of a MIP sensor was portrayed as reliable method for quantifying DA from biological samples, like diseases associated with elevated DA levels.

Recently, Saheed et al. [207] introduced a novel method for DA detection involving the electrodeposition of poly(2,4,6-trihydroxybenzaldehyde) (PTG) film on a GCE substrate. This work contributes to the ongoing advancements in electrochemical DA sensors by introducing a unique material. The subsequent electroanalytical evaluation of the PTG-modified GCE (PTGCE) revealed its capability for DA detection, exhibiting a favorable linear dynamic range of 0.70 to 19.48 μM and a low LOD of 0.64 μM. These results suggest that PTGCE exhibits a linear response, enabling effective quantification of DA within the concentration range of 0.70–19.48 μM. Moreover, the sensor demonstrated exceptional selectivity, particularly noteworthy in the presence of common interferences such as UA and AA, with well-separated peaks at 160 mV and 200 mV, respectively. This selectivity is crucial for reliable DA detection in real samples, where co-existing biomolecules pose challenges. The PTGCE’s performance was further bolstered by its good reproducibility in current response for DA, indicating consistent sensor behavior across measurements. Additionally, the impressive DA recovery rate of 99.70% suggests minimal DA loss during the detection process, facilitating the development of PTG-based electrochemical sensors for DA detection with promising analytical attributes, including good sensitivity, selectivity, reproducibility, and real-world applicability. Similarly, Shinde et al. [208] developed a Nb4C3Tx-MXene-silver nanoparticles (Nb4C3Tx-MXene-AgNP) fabricated laser-induced graphene (LIG) electrode. This design capitalizes on the unique properties of MXenes and AgNPs to achieve enhanced electrocatalytic activity for DA oxidation. The fabricated LIG electrode exhibited a significant improvement in its electrochemical performance, with the anodic current (Ipa) increasing from 150 μA to 330.4 μA, indicating the enhanced electrocatalytic activity of the modified electrode. This improvement was further validated by CV and CA techniques, demonstrating a high electrocatalytic response towards DA oxidation. The sensor displayed a linear detection range of 100 nM to 10 µM with a remarkably low detection limit of 1 nM. Additionally, the LIG-Nb4C3Tx-MXene-AgNPs electrode achieved an impressive sensitivity of 160.96 μA·nM^−1^·cm^−2^. Moreover, the sensor showcased excellent selectivity for DA detection, even in the presence of common interferents such as glucose, AA, and UA, effectively distinguishing dopamine and highlighting the designed electrode ability to function in complex biological environments. Table 5 summarizes various electrocatalysts reported towards sensing of DA [209,210,211,212,213,214,215,216,217,218,219,220,221,222,223,224].

### 4.6. Amino Acid Biosensors

Amino acids, recognized as the fundamental building blocks of proteins, play essential roles in various biological processes. These exist in two isomeric forms: L-isomers and D-isomers [225]. All L-isomers, with the exception of glycine, are nutritionally significant as they serve as protein building blocks and act as precursors for enzymes, hormones, and neurotransmitters. D-isomers are commonly found in bacterial cell walls and become particularly relevant during food processing or bacterial activity that alters protein structure, potentially impacting nutritional value and inducing health problems. The presence of D-amino acids in food can serve as an indicator of spoilage or adulteration. Detection of amino acids in the brain aids in understanding and diagnosing neurological disorders [225]. Traditional methods for amino acid detection are known to be complex, time-consuming, and cost-intensive, limiting their widespread applications. To overcome these barriers, biosensor methods have been introduced for their simplicity, speed, high sensitivity, and specificity in both in vitro and in vivo systems [226]. In recent years, electrochemical amino acid biosensors have successfully measured amino acid levels in various samples, including fruit juices, beverages, urine, and serum. These sensors have demonstrated good stability and reproducibility, with some sensors being reused up to 200 times over several months [226]. However, integrating nanostructures and microfluidic technologies able to further enhance sensitivity, selectivity, and miniaturization for various analytical applications, including food quality control, environmental monitoring, and clinical diagnostics [227].

**Figure 10:**
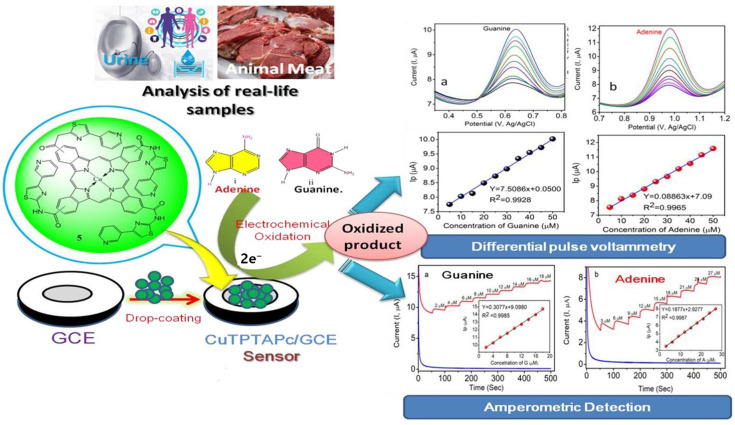
Copper–phthalocyanine molecule with amide linkages for simultaneous detection of adenine and guanine using an electrochemical method with improved sensitivity and superior detection for both nucleobases [228].

**Figure 11:**
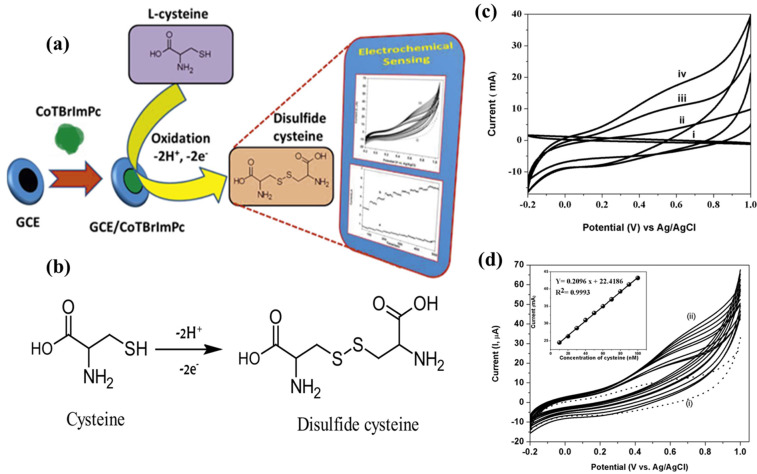
(**a**) CoTBrImPc/GCE for electrochemical biosensing of Cysteine, (**b**) oxidation mechanism of L-cysteine at GCE/CoTBrImPc, (**c**) CVs for (i) Bare GCE,(ii) Bare GCE with 50 nM L-cysteine, (iii) CoTBrImPc/GCE, and (iv) CoTBrImPc/GCE with 50 nM L-cysteine, (**d**) CVs for different L-cysteine concentration and plot of cathodic peak current vs. L-cysteine concentration [229].

In a recent study, Shantharaja et al. [228] synthesized a novel amide linkage, 4-(pyridine-3-yl)-4,5-dihydro-1,3-thiazol-copper phthalocyanine (Cu^II^TPTAPc), as shown in Figure 10. The Cu^II^TPTAPc film was deposited on GCE and utilized for the simultaneous detection of guanine (G) and adenine (A). The GCE/Cu^II^TPTAPc electrode exhibited a better voltametric range between 5 to 35 µM. Furthermore, DPV showed a linear response in the range of 5 to 70 µM with a LOD of 1.42 µM for both G and A. The CA method also displayed a linear response in the range of 2 to 18 µM and 3 to 27 µM for G and A, respectively, with LODs of 0.66 µM and 1.00 µM. The proposed sensor exhibited high selectivity for nucleic acid bases, as co-existing molecules did not respond during CA analysis. The designed electrode was successfully employed for the analysis of real sample i.e., urine. However, the real samples, like blood, contain many other interfering molecules, hence an ideal detection method that cannot be affected by their presence needs to be developed. Similarly, Keshavananda prabhu et al. [229] synthesized the dark blue colored cobalt (II) tetra[4-(2-{(E)-[(4-bromophenyl)imino]methyl}phenoxy)] phthalocyanine (CoTBrImPc), exhibiting promising electrocatalytic activity towards L-cysteine detection. Immobilized on GCE, it demonstrated excellent performance with a low detection limit of 3 nM and high sensitivity of 2.99 μA·nM^−1^·cm^−2^, with a linear response range from 10 to 100 nM, showcasing potential applications in L-cysteine sensing as shown in Figure 11. Shantharaja et al. [230] synthesized the novel *pyrazolopyrimidinium* analog bearing cobalt (II) phthalocyanine polymer (poly-CoTPzPyPc) and characterized its sensing ability for L-arginine. The polymer film displayed a linear response to L-arginine concentration in the range of 10–100 μM with a LOD of 2.5 μM. Rotating disc electrode experiments confirmed a two-electron transfer process during L-arginine oxidation, and chronoamperometric studies showed catalytic response for L-arginine in the range of 2 to 60 μM with a limit of detection of 0.6 μM. The poly-CoTPzPyPc film exhibited excellent selectivity towards L-arginine in the presence of biomolecules, and the sensor demonstrated good stability and satisfactory performance in real sample analysis of urine. These findings suggest that poly-CoTPzPyPc holds promising potential for the detection and monitoring of L-arginine in biological samples. Due to their structural similarities with other amino acids, electrochemical methods face bottlenecks such as isolating actual signal (e.g., adenine/guanine/L-cysteine/L-arginine) from similar molecules, electrode surface fouling, weak inherent response, low sensitivity, and limited portability in current setups pose challenges in commercialization. Hence, researcher’s emphasize specialized electrode materials, molecularly imprinted electrochemical sensor, disposable screen-printed graphite electrode, enzyme-based sensors, and integrating electrochemistry with other techniques. Overcoming these obstacles leads to reliable, sensitive, and portable methods for amino acids detection.

**Table 6:** Overview of various macrocycles and their hybrids as amino acid biosensors.

Electrocatalyst Material	Linear Range	Sensitivity	LOD	Ref.
Poly(CoTBrImPc)	10–100 nM	2.99 μA·nM^−1^·cm^−2^	3 nM	[229]
poly-CoTPzPyPc	10–100 μM	-	2.5 μM	[230]
RGO-pTACoPc	0.05–2.0 μM	10.19 nA·nM^−1^·cm^−2^	0.018 μM	[231]
Pd-NP/CPE	0.6 to 112 μM	-	200 μM	[232]
Urease-based ion-selective field effect transistors (ISFET) biosensor	0.5–148 µM	5.4 nA·µM^−1^	-	[233]
Graphite Teflon electrode-modified L/D-arginine oxidize	100–1000 μM	-	160 and 33 μM	[234]
Arginine deiminase (ADI)/PANI/Nafion/Pt-SPE	3–200 μM	-	1 μM	[235]
PbPc	1–50,000 μM	-	1 μM	[236]
L-tryptophan	5–150 μM	-	1.73 and 5.78 μM	[237]
Screen-printed diamond electrode	1–194 μM	-	0.62 μM	[238]
Functionalized MWCNT	0.7 nM–200 μM	-	0.16 nM	[239]
Carbon black functionalized with syringic acid	20–100 μM	-	0.639 μM	[240]
Iron tetrasulphonated phthalocyanine-decorated MWCNT	10–200 μM	-	1 μM	[241]
Co(II)-phthalocyanine	2.6–200 μM	0.78 μA·µM^−1^·cm^−2^	4 μM	[242]
MWCNT and molecularly imprinted polymer	0.002–100 μM	-	0.001 μM	[243]
4-amino-3-hydroxyl-1-naphthalenesulphonic acid/rGO based polymer	0.5–200 μM	0.0451 μA·μM^−1^	0.31 μM	[244]
rGO-hemin-Ag	0.1–1000 μM	-	0.03 μM	[245]
Molecularly imprinted polymer/rGO	0.1–400 μM	-	0.046 μM	[246]

Note: PdTAPc/MWCNT—Tetraamino-substituted palladium phthalocyanine modified multiwalled carbon nanotubes; Poly(CoTBrImPc)—Cobalt (II) tetra[4-(2-{(E)-[(4-bromophenyl)imino]methyl}phenoxy)] phthalocyanine; RGO-pTACoPc—Cobalt phthalocyanine/reduced graphene oxide;Pd-NP/CPE — Carbon paste electrode palladium nanoparticles; —Lead phthalocyanine; Functionalized MWCNT—Functionalized multiwalled carbon nanotubes; rGO-hemin-Ag—Reduced graphene oxide with silver; Molecularly imprinted polymer/rGO—Molecularly imprinted polymer/reduced graphene oxide.

Hernández et al. [242] successfully constructed a L-cysteine electrochemical sensor to assess L-cysteine levels in a complex embryo cell. L-cysteine was electro-analytically determined using a disposable screen-printed graphite electrode modified with Co(II)-phthalocyanine i.e., CoPc-SPE. Cyclic and square wave voltammetry (SWV) demonstrated strong electrocatalytic activity for L-cysteine oxidation at neutral or basic pH levels. The SWV response of L-cysteine showed a linear range of 2.6 to 200 μM, a low LOD (4 μM, S/N = 3), and a sensitivity of 0.78 μA·cm^−2^·μM^−1^. The reproducibility and repeatability of the developed electrode had low coefficients of variation (3% and 0.4%, respectively). It was also evaluated for interferences such as naturally occurring amino acids, cystine, and cysteic acid. Similarly, Wu et al. [243] created a molecularly imprinted electrochemical sensor for tryptophan (Trp) by drop-coating an imprinted chitosan film onto GCE modified with MWCNTs (MIP-MWCNTs). CV revealed improved electrochemical properties at MIP-MWCNTs modified electrode. The MIP-MWCNTs/GCE had a higher Trp oxidation current than the uncovered GCE, indicating improved electron transfer kinetics and strong Trp selectivity. Optimization revealed a linear relationship between Trp oxidation peak current and concentrations ranging from 2.0 nM to 0.2 μM, 0.2–10 μM, and 10–100 μM with a low LOD of 1.0 nM (S/N = 3). Lata et al. [247] developed a highly sensitive amperometric L-amino acid biosensor. Additionally, covalent immobilization of L-amino acid oxidase (LAAO) onto a hybrid film comprising nickel hexacyanoferrate (NiHCNFe), polypyrrole (PPy), and carboxylated MWCNTs (CMWCNT) electrodeposited onto a GCE surface. At pH 7.5, 35 °C, and polarization set at 0.15 V vs. Ag/AgCl, the response materialized in 5 microseconds. In the range of 0.5 μM to 100 mM, the biosensor demonstrated linearity for L-phenylalanine concentration. Its sensitivity was found to be 79.31 nA·cm^−2^·μM^−1^, and its detection limit was 0.5 μM (S/N = 3). Similarly, Pei et al. [248] used 10 wt% polyaniline/CuGeO_3_ for electrochemical determination of L-cysteine. The modified electrode showed two pairs of CV peaks for L-cysteine detection. The anodic peaks for L-cysteine were 0.24 and 0.07 V, and the modified electrode showed linear growth with increasing L-cysteine concentration within the range of 0.001–2 mM in the scan rates ranging from 25 to 200 mV/s. The modified electrode demonstrated high reproducibility, stability, and low detection limits (1.7 and 0.44 μM in CVP1 and CVP2, respectively). The combination of polyaniline and CuGeO_3_ nanowires improved the electrochemical detection of L-cysteine. The hybrid electrode showed two types of partially reversible electrochemical CVP. These peaks correspond to L-Cys and L-cystine’s oxidation-reduction processes, as well as their adsorption and desorption processes. Likewise, Ramya et al. [249] investigated the development of a novel sensor for L-Tyrosine (L-Tyr) detection using electrochemically synthesized molecularly imprinted polymers (MIPs). L-Tyr is a crucial amino acid affecting melanin, DA, and insulin resistance. Prior L-Tyr detection methods employed expensive materials and complex procedures. The MIPs were synthesized via pyrrole electro-polymerization on GCE modified with functionalized MWCNTs. The sensor displayed exceptional reproducibility, stability, and a broad linear detection range (1–2000 μM). The LOD and quantification were found to be 0.4 μM and 1.47 μM, respectively. Validation using human blood and serum samples yielded satisfactory recovery, demonstrating potential for future multi-analyte detection. The development of these advanced electrochemical sensors faces several issues. Optimizing the properties of specialized electrode materials and molecularly imprinted polymers for target-specific detection remains a challenge. Achieving a balance between cost-effectiveness and performance in disposable screen-printed graphite electrode necessitate continuous research efforts. Furthermore, integrating electrochemical methods with synergistic analytical techniques provide comprehensive data and presents optimization to overcome integration hurdles. Finally, optimization of the entire analytical system, encompassing sample preparation, signal processing, and data analysis, is important for real practical applications. Addressing these ongoing research areas will lead to the development of durable, easy-to-use, and multifunctional electrochemical sensors. Table 6 summarizes various biosensor catalysts reported so far for the detection of various amino acids [182,229,230,231,232,233,234,235,236,237,238,239,240,241,242,243,244,245,246].

### 4.7. Cholesterol Biosensors

Animal cells produce cholesterol, a common sterol, via biosynthesis [250]. It functions as a precursor for bile acids, vitamin D, and steroid hormones, and it is transported by lipoproteins in the blood stream [251]. It is primarily categorized as (i) triglycerides, (ii) low density lipoprotein (LDL), also referred to as “bad” cholesterol, and (iii) high density lipoprotein (HDL), also known as “good” cholesterol. It exists in chemical form in human body fat [252]. Different roles are played by each cholesterol type in the blood stream and metabolism. Nonetheless, the correlation between optimal cholesterol levels and a number of pathological conditions, including hypothyroidism, nephrotic syndrome [253], stroke, heart attacks [254], diabetes [255], and liver diseases, suggests that maintaining optimal cholesterol levels is essential for preventing cardiovascular diseases (CVDs) [256]. CVDs are one of the top three diseases that cause a high mortality rate and death. Thus, maintaining ideal cholesterol levels is essential for protecting human health. Therefore, it is essential to ensure optimal health and facilitate accurate diagnoses to implement early detection, proactive prevention, and effective health management [257]. Additionally, the effective and early detection techniques for cholesterol involves mass spectroscopy, fluorimetry, and HPLC [258]. Recently, electrochemical methods for biosensors, have received attention due to their low cost, low detection limit, high sensitivity, quick recovery time, and specificity [259]. Their small size and ability to work with microfluidic technologies make them extremely promising for point-of-care and personalized medicinal applications [260]. They could potentially revolutionize the management of cardiovascular healthcare by enabling real-time cholesterol monitoring [261]. This method uses two distinct techniques, including enzymatic and non-enzymatic methods, to detect cholesterol. An enzymatic reaction converts cholesterol to free cholesterol (cholest-3-one) by the action of cholesterol oxidase (ChOx), which releases hydrogen peroxide (H_2_O_2_) as a byproduct [262]. Nevertheless, there are still issues with enzymatic sensing, such as high cost, long-term stability, storage, and degradation of the enzymes, among others. From this angle, scientists focused on non-enzymatic sensing because of its ease of synthesis, great stability, repeatability, and lack of oxygen constraint. The challenge still lies in selecting an appropriate electroactive material, with respect to selectivity, sensitivity to detect up to pico-molar analyte concentrations, and limited electronic communication between material active sites. Diverse metal/metal oxide nanoparticles were investigated for effective and trustworthy cholesterol biosensors. For instance, Anh et al. [263] designed optimized silver nanoparticles with zinc oxide nanorods (Ag/ZnO-NRs) based biosensor in a phosphate-buffered saline solution for the electron mediation in an electrochemical reaction via cholesterol oxidation at the allylic position. With a superior sensitivity of 4.2 µA·mM^−1^·cm^−2^ for ZnO-NRs and an astounding 135.5 µA·mM^−1^·cm^−2^ for the Ag/ZnO-NRs composite, the proposed biosensor demonstrated a linear detection range of 1–9 mM. Furthermore, the detection limit was impressively as high as 0.184 mM. In addition, it exhibited outstanding stability, repeatability, and reusability for practical applications. Similarly, Li et al. [264] reported a simple method of fabricating porous tubular Ag nanoparticles onto a GCE electrode for use as a cholesterol biosensor. With a linear detection range of 2.8 × 10^−4^ M to 3.3 × 10^−2^ M and a LOD of 1.8 × 10^−4^ M, the hybrid electrode (Ag into cadmium sulfide) demonstrated significantly higher electrocatalytic activity towards cholesterol oxidation compared to Ag. The promising properties of porous tubular Ag for advanced biosensing applications are highlighted by its remarkable stability, reproducibility, and low interference. However, challenges exist in this method, due to its dependency on hazardous CdS for fabrication, and hence promising fabrication studies are needed to assess the sensor’s long-term stability and reusability for practical applications. On the other hand, metal oxides have high conductivity, exhibit cytotoxicity or instability in biological environments, and their interaction with cholesterol may not directly translate into a significant electrical response. They also exhibit non-specific binding and signal interference. Since polymer-based materials have shown promise in overcoming these obstacles, a beta-cyclodextrin (β-CD) based optic-microfiber biosensor was recently developed by Zifan et al. [265] to detect cholesterol concentrations. To form an inclusion complex through cholesterol reaction, β-CD immobilizes on the fiber surface as an identifying substance. The suggested sensor translates the change in surface refractive index (RI) into a macroscopic wavelength drift in the interference spectrum when the RI change is caused by capturing the complex cholesterol (CHOL). With a low-temperature sensitivity of −0.019 nm/°C, the microfiber interferometer exhibited a low RI sensitivity of 1251 nm/RIU. This sensor has a sensitivity of 12.7 nm/(mM) in the low concentration range of 0.001 to 0.05 mM and can quickly detect cholesterol in the concentration range of 0.001 to 1 mM. This biosensor demonstrated high sensitivity and selectivity, making it suitable for biomedical use. Though the β-cyclodextrin-based biosensor shows promise with high sensitivity and selectivity for cholesterol detection, challenges remain. Specificity towards other biomolecules in real samples, long-term stability of the coating, sensor reusability, and biocompatibility for in vivo use need investigation.

**Figure 12:**
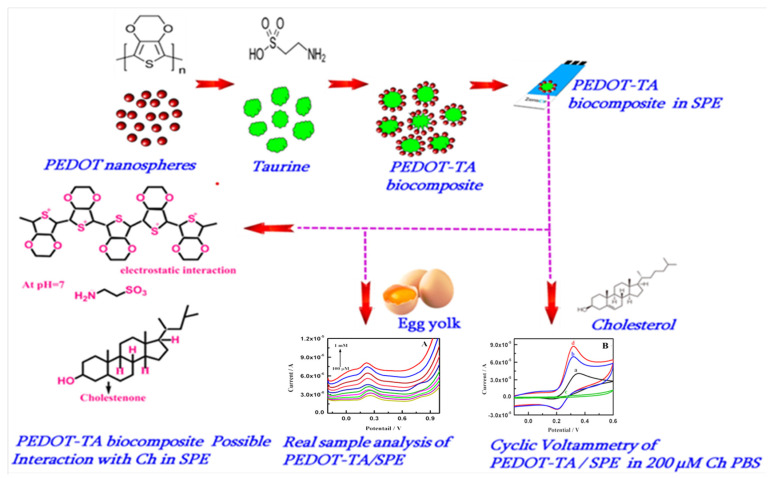
Schematic presentation of PEDOT-TA hybrid material for cholesterol sensing mechanism. Adapted with permission from ref. [266], copyright 2023, Elsevier.

**Figure 13:**
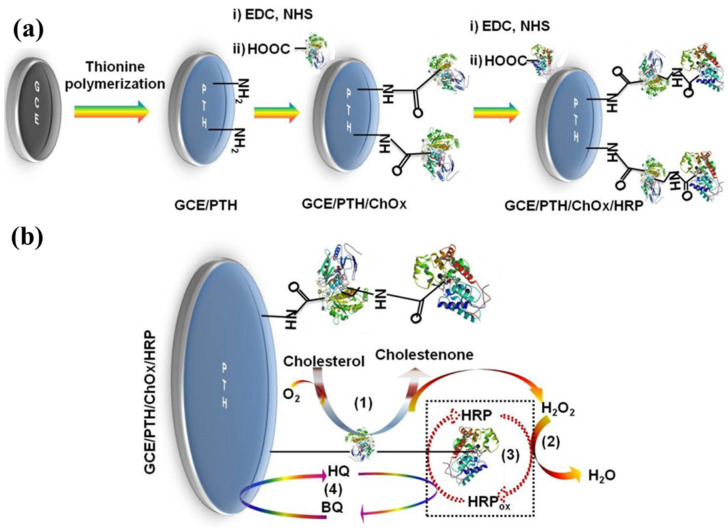
(**a**) GCE/PTH/ChO_x_/HRP biosensor fabrication and reaction process, (**b**) protein structures for ChOx and horseradish peroxidase. Adapted with permission from ref. [268], copyright 2023, Elsevier.

Additionally, Thivya et al. [266] investigated the use of poly(3, 4-ethylenedioxythiophene) (PEDOT) coated with taurine (TA) on a screen-printed electrode (SPE) as a non-enzymatic electrochemical method for the detection of cholesterol, as shown in Figure 12. High stability and dispersity were achieved through the electrostatic interaction promoted by TA sulphonic acid. Using a linear response (3 µM to 1 mM) in both cyclic and square wave voltammetry, the PEDOT/TA composite demonstrated a lower detection limit of 0.95 µM. To demonstrate the practical application of egg yolk with ch for the modified PEDOT/TA/SPE, square wave voltammetry (SWV) was performed in a 0.1 M PBS solution (pH 7.0) showed in Figure 12A. The CV behavior shown in Figure 12B (a) bare SPE, (b) PEDOT/SPE (poly(3,4-ethylenedioxythiophene) modified SPE), (c) TA/SPE (thionine adsorbed SPE), and (d) PEDOT-TA hybrid bio-composite modified SPE. Similarly, for the electrochemical detection of cholesterol, Yang [267] and colleagues used methylene blue (MB) and calix[6]arene functionalized graphene (CX6–Gra). The technique demonstrated a low LOD of 0.20 μM and a linear response range of 0.50 to 50.00 μM for cholesterol. The versatility was further demonstrated by the analysis of serum samples and the possibility of expanding the analysis to include other non-electroactive species. The internal bonding relation between the CX6 complexation and cholesterol was revealed by the molecular modelling calculations. By exhibiting the lowest binding free energy of −8.01 kcal·mol^−1^, this 1:1 host–guest stoichiometry guarantees the effective and selective detection of cholesterol, overcoming interference from common species. Similarly, Rahman et al. [268] designed a cholesterol sensor by immobilizing cholesterol oxidase (ChOx) and horseradish peroxidase (HRP) onto a poly(thionine) (PTH)-modified GCE (GCE/PTH/ChO_x_/HRP), as shown in Figure 13. The modified electrode exhibited excellent electrocatalytic activity by reducing H_2_O_2_, which was obtained from cholesterol on enzymatic reaction of ChOx. The biosensor achieved a linear detection range of 25–125 µM for cholesterol analysis, with a LOD of 6.3 µM and good sensitivity of 0.18 µA·cm^−2^·µM^−1^. This sensor displayed excellent reproducibility, high sensitivity, and resistance to interferences, making it promising for real sample analysis.

More recently, Eghbali et al. [269] developed a non-enzymatic cholesterol biosensor and utilized a MIP-based on electropolymerized para-phenylenediamine (p-PD) on copper foam (CF) modified with a Pt/CuO dual-core nanohybrid. The biosensor achieved a detection limit of 0.035 μM, a sensitivity of 157.85 µA·µM^−1^·cm^−2^, and a linear range of 0.4 to 6 μM. Notably, density functional theory (DFT) was utilized to optimize the MIP design, and the effect of sensor loading time on cholesterol detection was investigated for the first time. This research highlights the potential of CF/CuO/Pt/CHO-MIP nanocomposite as a promising platform for selective and sensitive cholesterol biosensors. Similarly, Murugesan et al. [270] utilized a polyaniline (PANI) and its nanocomposites (NCs) with ZnO nanoparticles (NPs). The study explored the impact of ZnO loading (2%, 3%, 5%, 8%) and dopant type (camphor sulfonic acid, HCl) on sensor performance. Electrochemical analysis revealed improved behavior with increased ZnO content and for HCl-doped PANI NCs. PANI/ZnO 8% NCs with both dopants showed promising response for cholesterol sensing. The sensor exhibited a sensitivity of 0.38 mA·mM^−1^·cm^−2^ and a LOD of 0.30 mM in PBS buffer with a linear range of 0.75–1.5 mM. While both the CF/CuO/Pt/CHO-MIP and PANI/ZnO nanocomposites exhibited high potential cholesterol detection level, they still have limitations for ideal compound realization. The CF/CuO/Pt/CHO-MIP sensor requires studies on long-term stability, in vivo performance, and reusability. The PANI/ZnO sensor needs to improve selectivity for complex biological samples and optimize sensor loading time. Additionally, a simpler fabrication process for PANI/ZnO would be ideal. These aspects should be addressed in the pursuit of an optimal material for cholesterol biosensors. Table 7 summarizes various electrocatalysts reported so far for sensing of cholesterol [271,272,273,274,275,276,277,278,279,280,281].

### 4.8. Cancer Biosensors

**Figure 14:**
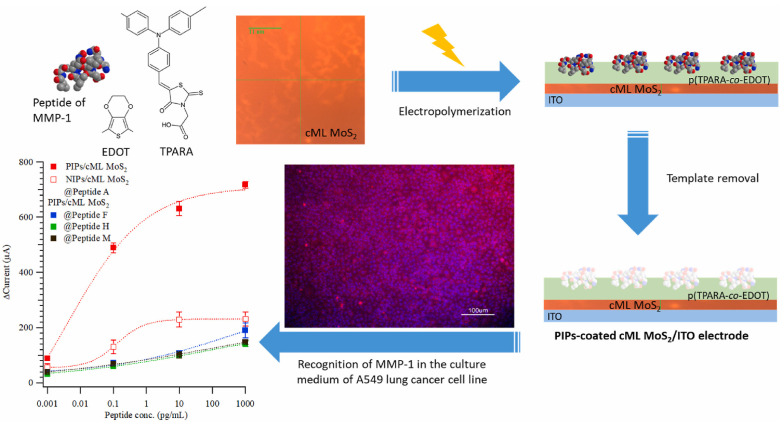
Design and selective detection of MMP-1 using peptide-imprinted polymer on molybdenum disulfide electrode for A549 lung cancer cell [295]. Adapted with permission from ref. [295], copyright 2024, Elsevier.

Cancer continues to be the leading cause of death worldwide [282]. Despite significant investments in efficient developments, cancer mortality remains high [283]. According to the World Health Organization, there were approximately 10 million deaths and 19.3 million new cases worldwide in 2020 (Cancer (who.int)). The death rate has dropped over the past few years as a result of early cancer detection benefits from eliminating cancer cells, as well as increased knowledge of better diagnostic methods [284], cutting-edge tumor biology, and enhanced treatments [285]. Since many anticancer drugs are hydrophobic and do not have high affinity for cancer cells [286], their ability to distribute poorly on the cell surface has limited the use of cytotoxic drugs in current therapeutic regimes [287]. As per this paradigm, cancer pathologies include intense radiation, chemotherapy [288], and hormonal therapy medications that frequently result in the death of healthy cells and cause toxicity [289]. It is therefore necessary to create a more potent chemotherapy technique. “Smart nanomaterials,” including liposomes, micelles, dendrimers, and polymeric nanoparticles, have demonstrated a range of uses in biosensing, controlled drug delivery [290], nanomedicines, wastewater treatment, catalysis, and other areas [291]. These nanomaterials have shown promise in cancer therapy by enabling the development of targeted drug delivery systems or active-intracellular delivery into cancerous cells, thereby overcoming all limitations (poor cell penetration, uncontrolled drug release, and slow bioavailability) [292]. Additionally, by incorporating NPs, nanocomposites improve the characteristics of materials like metals, ceramics, and polymers. A variety of nanocomposites have been developed, including metal matrix nanocomposites (Cu, Mg, Ti), ceramic matrix nanocomposites (Si_3_N_4_/SiC, Al_2_O_3_/SiC, MgO/SiC), and polymer matrix nanocomposites (e.g., polyethylene glycol (PEG), polyvinyl alcohol (PVA), chitosan) [293]. However, because of their special qualities, such as high fluorescence brightness, high surface area, tunable fluorescence quantum yield, biocompatibility, fast emission rate, low cytotoxicity, and photostability, nanomaterials significantly improve the sensitivity and specificity of designed biosensors for detecting cancer biomarkers [286]. This results in more accurate diagnostics with the early and reliable detection of cancer-related molecules. Furthermore, the incorporation of polymeric nanocomposites into biosensor devices improves the signal-to-noise ratio and allows for the comprehensive diagnosis of early-stage cancer, which is essential for prompt intervention, leading to better patient recovery and outcomes. As a result, the biocompatible nature of polymeric nanocomposite guarantees that there will be little interference with biological frameworks. It is possible to modify the surface of the nanocomposite to improve its sensitivity and selectivity in the detection of particular DNAs, miRNAs, enzymes, and proteins, which in turn allows for timely and precise diagnosis. According to reports, whole cancer cells with LOD values of approximately 10 cells, as well as DNAs, miRNAs, proteins, enzymes, and neurotransmitters with LOD values as low as 0.5 fM [294], can be detected. Similarly, Lee et al. [295] discovered a novel electrochemical biosensor for detection of matrix metalloproteinase-1 (MMP-1), a potential biomarker for lung cancer. The research group designed the peptide-imprinted poly(triphenylamine-co-rhodamine) (PTARA) film-doped molybdenum disulfide (MoS_2_) monolayer (cML) as depicted in Figure 14. This approach helped the imprinting process to enhance the target selectivity. The PTARA-functionalized MoS_2_ cML electrode showed good response towards MMP-1 with a lower concentration of 1.0 pg/mL. This response was 1.4 times greater compared to non-imprinted electrodes. Notably, the MoS_2_ cML-coated PTARA extended the sensor’s detection range from 1.0 pg/mL to 10.0 pg/mL, which offered broader MMP-1 concentration analysis. Furthermore, the sensor was used to measure MMP-1 concentration in A549 lung cancer cell culture medium. The obtained value of 800 ng/mL is in good agreement with higher accuracy of 95 ± 5%, and it can replace the commercially available ELISA kit in respect to sensor effectiveness.

**Figure 15:**
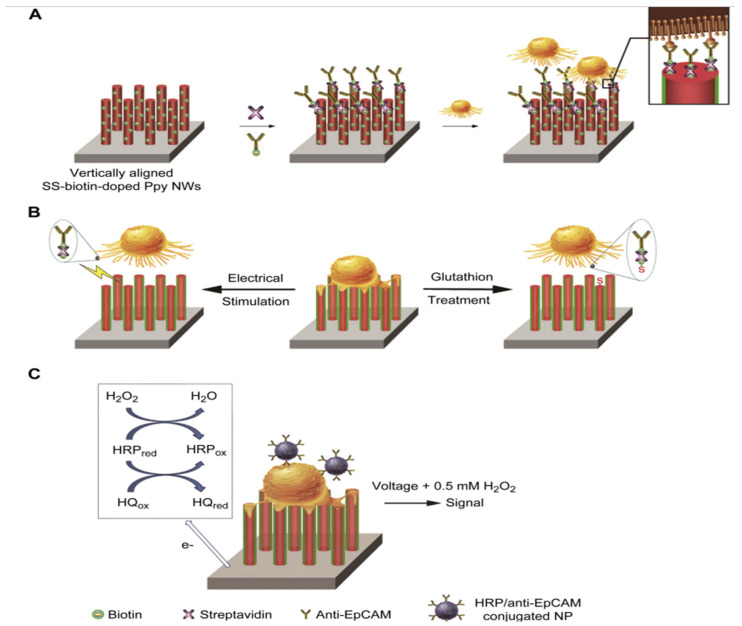
(**A**) Anti-EpCAM antibodies immobilized on biotin-doped polypyrrole nanowires for detection of cancer cells, (**B**) glutathione-triggered controlled drug release, (**C**) cancer cell detection using nanoparticle amplification strategy for electrochemical biosensing [299]. Adapted with permission from ref. [299], copyright 2024, Elsevier.

Electrochemical methods have drawn the most attention for sensing of various analytes due to their ability to provide rapid and continuous detection. However, broad adoption for practical applications remains a challenge. The recent study by Amani and group [296] described an electrochemical immunosensor for the tumor marker CA 15-3. A composite of rGO and copper sulfide (CuS) deposited on a screen-printed graphite electrode served as the detection material. The sensor exhibited efficient catechol oxidation, with optimal performance at 0.16 V. Following modification with anti-CA15-3 antibody, CA 15-3 binding led to a decrease in the catechol response, which displayed a linear range of 1.0–150 U mL^−1^ CA 15-3, a detection limit of 0.3 U mL^−1^, and a sensitivity of 1.88 µA·µM^−1^·cm^−2^. However, further investigation is needed to ensure selectivity towards CA 15-3 in real serum samples, where other biomolecules might interfere and the reusability of the sensor requires evaluation for practical applications. Li et al. [297] designed a bovine serum albumin with polyethyleneimine (BSA-PEI) composite material for cancer antigen 125 (CA125) detection, and portrayed the antifouling performance in complex biological environments. The composite exhibited strong hydrophilicity with near neutral charge, and showed minimal unwanted interactions with other materials and charged biomolecules. Furthermore, an electrochemical biosensor for cancer antigen 125 (CA125) detection was constructed utilizing MXene’s high conductivity and the antifouling properties of BSA-PEI. The biosensor achieved a low detection limit of 2.6 mU/mL in human serum. It highlights the potential of BSA-PEI for developing effective antifouling biosensing devices. Furthermore, blood serum samples have a protein-rich environment that encourages the buildup of undesirable biomolecules on the biosensor surface, which impairs its functionality and eventually results in decreased sensitivity and stability. Addressing the biofouling effect has become a central focus for researchers [294]. For example, polymeric nanocomposites, such as Hb, Pcs, porphyrins, corroles, and molecularly imprinted polymers (MIP), are described as dependable materials to achieve the highest sensitivity and selectivity, and provide drug efficacy at various sites with quick, accurate detection. Furthermore, they have the potential to reliably image cancer cells, monitor angiogenesis and metastasis, and assess the efficacy of anticancer chemotherapy agents. Particularly, the detection technologies for chemotherapy demonstrated potential in lowering side effects, boosting biocompatibility, and enhancing therapeutic efficacy. In recent research, Ribeiro et al. [298] created a synthetic receptor film called electrically conductive poly(toluidine blue) that is specifically designed to detect the breast cancer biomarker carbohydrate Antigen 15-3 (CA 15-3). With a linear detection range spanning two orders of magnitude (0.10–100 U mL^−1^), the polymeric receptor demonstrated a ~12-fold higher binding affinity towards CA 15-3. The sensor responds predictably to increasing CA 15-3 concentrations, and its lower LOD was achieved through DPV in both buffer and diluted serum. With the potential for actual practical applications, this method for CA 15-3 screening is therefore simple, quick, and affordable. However, further investigation is needed to improve the selectivity towards CA 15-3 in complex biological samples (serum). Additionally, long-term stability of the imprinted film and its reusability need to be addressed for practical applications. Similarly, Hong et al. [299] designed conductive disulfide-biotin-doped polypyrrole nanowires (SS-biotin-Ppy NWs) for cancer cell detection, as shown in Figure 15. The SS-biotin-Ppy NWs were efficiently employed for surface bound monoclonal antibody, and they exhibited controlled drug release ability through glutathione (GSH). Furthermore, the strategy utilized horseradish peroxidase (HRP) and anti-EpCAM conjugated nanoparticles (HRP/anti-EpCAMPpy NPs) which demonstrated exceptional sensitivity and specificity for captured cancer cell identification. Signal amplification resulted from the enzymatic reduction of hydrogen peroxide on the nanowires, which lead to a highly amplified response with a wide detection range of 10–10^4^ cells and a remarkably low detection limit of just 10 cells.

**Figure 16:**
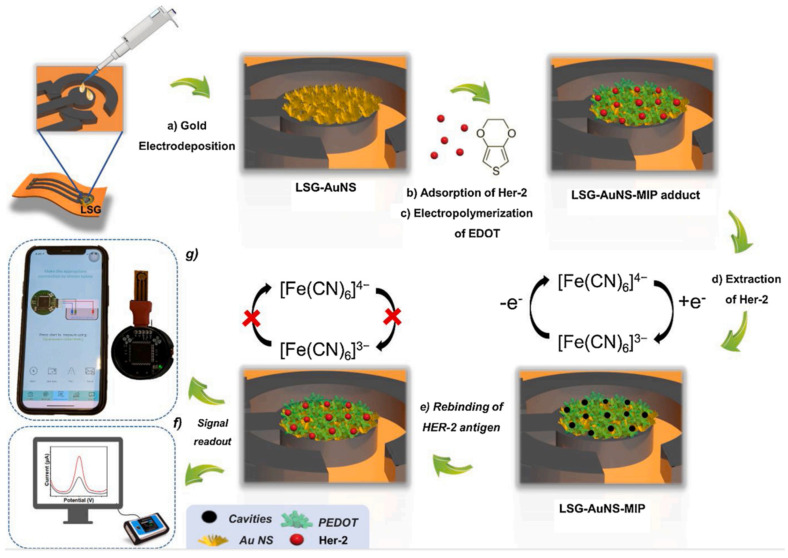
Schematic of the LSG-AuNS-MIP biomimetic sensor, (**a**,**b**) AuNS electrodeposition, (**b**) Her-2 adsorption, (**c**,**d**) MIP formation, (**e**) Her-2 rebinding, and (**f**,**g**) signal response. Adapted with permission from ref. [302], copyright 2024, Elsevier.

Peng et al. [300] designed the polycrystalline Cu^2+^-NMOFs assembled with a DNA bridge electrochemical biosensor for sensitive detection of mesenchymal cells. The fabricated electrode was antibody-specific to vimentin, and a mesenchymal biomarker on the electrode surface. Cholesterol-labeled functional nucleic acids on the Cu^2+^-NMOFs was utilized for the dual purposes of anchoring the probes to the captured mesenchymal cells through the cell membrane as well as bringing them close to the electrode surface. This strategy enabled sensitive detection of mesenchymal cancer cells in the range of 2 × 10^1^ to 10^4^ cells mL^−1^ with 5 cells mL^−1^ detection limit. Furthermore, the sensor has high selectivity and anti-interference capability, and highlighted its potential utilization for future clinical cancer diagnostics. Song et al. [301] developed an organic electrochemical biosensor for in situ detection of cancer cells in blood. This sensor was fabricated by carboxyl graphene mixed with PEDOT:PSS. The utilization of carboxyl graphene modulates the cancer cell morphology, and acts as the differentiator from normal blood cells. Increasing the carboxyl graphene concentration (0–5 mg mL^−1^) led to an increase in cancer cell surface area (218 μm^2^ to 530 μm^2^). The organic electrochemical biosensor effectively detected the changes in cell morphology. The presence of negatively charged cancer cells induced the positive voltage shift (nearly 40 mV for spherical cells), while the increased surface area resulted in the negative shift. This strategy enabled the sensor to identify deformable cells during capture, potentially differentiating the cancer cells. Similar to this, Lahcen et al. [302] created molecularly imprinted polymers (MIPs) and nanostructured gold (AuNS) for the detection of human epidermal growth factor receptor 2 (Her-2) as a breast cancer biomarker, as indicated in Figure 16. The sensitivity of this sensing was as low as 0.43 ng/mL, and it demonstrated a remarkable ability to detect Her-2 over a concentration range of 1–200 ng/mL. Additionally, the biomimetic sensor demonstrated a strong recovery of Her-2 in undiluted human serum samples and exhibited high selectivity towards Her-2 among interfering molecules. Her-2 was successfully detected using the LSG-AuNS-MIP sensor. Sensing of different cancer cells using various macromolecule-based hybrid composites reported in the literature has been summarized in Table 8 [303,304,305,306,307,308,309,310,311,312,313,314,315].

## 5. Conclusions, Challenges, and Future Directions of Macromolecule-Based Hybrids in Biosensor Applications

In summary, this manuscript points out the remarkable potential of hybrids derived from macrocycles and macromolecules to assist in the detection of crucial biomolecules. These hybrids exhibit excellent durability, accuracy, and sensitivity while diligently overcoming a variety of obstacles, such as redox behavior, cost-effectiveness, and synthetic complexities. However, more development is still required to reach unmatched sensitivity and extremely low detection limits, especially when dealing with complex biological matrices. Macromolecule synthesis requires careful selection of reactants with flexible functional groups in order to achieve sensor robustness in the presence of interferents and environmental fluctuations. Significant measures must be adopted in order to facilitate real-time sensing of biomolecules in different medical conditions, which is a basic requirement for facilitating the development of portable, wirelessly connected devices. Once biocompatibility and stability issues are resolved, the opportunity of hybrid sensor functionalization for multi-analyte detection holds innovative advantages over diagnostic assays, creating the way to customized healthcare. Analyte–electrode interface and sensor variability optimization are necessary to address ongoing issues with biocompatibility, stability, and reproducibility. Investigating new materials, nanostructures, and coatings offers viable paths to improve accuracy and efficiency. To further advance biosensor technology, the incorporation of hierarchical nanostructures appears to be a promising path. Biosensor performance can be greatly improved by these nanostructures because they have unique benefits like increased surface area and improved signal transduction. Through the fusion of hierarchical nanostructures to form hybrids based on macromolecules, scientists can explore novel possibilities for achieving superior sensitivity, extremely low detection limits, and improved noise control in biosensing applications. In addition, standardization initiatives and regulatory cooperation are essential for effective clinical collaboration. To fully utilize biosensing platforms and improve global health outcomes, a multidisciplinary approach integrating expertise in material sciences, biotechnology, and engineering is crucial.

## Figures and Tables

**Table 1:** Macrocycle/carbon nanoparticles-based hybrid composites as glucose biosensors.

Electrocatalyst Material	Linear Range	Sensitivity (µA·mM^−1^·cm^−2^)	LOD	Ref.
GR/CuO NPs nanocomposites	1 mM to 8 mM	1065	1 µM	[89]
GCE/GO-Ph-AuNPs-CP/Gox	0.3 to 20 nM	42	-	[92]
Nickel Cobalt Alloy Nanoparticles Anchored on Polypyrrole/Reduced Graphene Oxide	0.5–4.1 µM	153.5	0.17 µM	[93]
NiCo_2_N/N-GR core–shell	0.5 µM to 4.1 mM	1803	50 nM	[94]
GCE-SWCNT/rGO/CoPc	0.0003–0.50 mM	992.4	0.12 μM	[95]
AlPc/MWCNTs	50–500 µM	0.058	2.5 nM	[96]
Ni_2_Co_1_-BDC on GCE	0.5–2899 µM	3925	0.29 µM	[97]
Dendritic gold nanostructures (DGNs) modified on graphite electrode	Up to 9.97 mM	-	0.059 mM	[98]
glucose oxidase (GOX) onto poly(3,4-ethylenedioxythiophene):4-sulfocalix[4]arene (PEDOT:SCX)/MXene	0.5–8 mM	-	22.5 µM	[99]
NiO NPs/polyaniline nanowire/GO composite	0.002–5.56 mM	376.22	0.5 µM	[100]
CuNPs/rGO core–shell nanocomposite	0.001–2 mM	150	0.34 µM	[102]
rGO/Ni(OH)_2_ nanostructures	15 µM to 30 mM	11.4	15 µM	[103]
PtPd-IL-rGO nanocomposite	0.1–22 mM	1.47	2 µM	[104]
Ni(OH)_2_/3D GF	1 µM to 1.17 mM	2.65	0.34 µM	[105]
GR/PtNi NPs	0.5–20 mM	30.3	2 µM	[106]
Cs/Cu-MOF/SPCE	2 to 1700 μM	1378.11	2 μM	[107]
CuO@CNTFs	up to 13 mM	~3000	1.4 μM	[108]
PVP–TiO_2_ NCs	0 to 13 mM	360.13	756.8 µM	[109]
MOF MnOx-CNFs	0 to 9.1 mM	80.6	0.3 μM	[110]
U-Shaped Microfiber with GOx	0–3 mg/mL	5.73 nm/(mg/mL)	0.17 mg/mL	[111]
PVP/GNs/NiNPs/CS composite	0.1 µM to 0.5 mM	103.8	30 nM	[112]

Note: AlPc/MWCNTs—Aluminum(III) Phthalocyanine-multiwalled carbon nanotubes; GCE-SWCNT/rGO/CoPc—Single walled carbon nanotubes on reduced graphene oxide with cobalt phthalocyanine; GCE/GO-Ph-AuNPs-CP/Gox—Graphene oxide with phenyl bridged gold nanoparticle using glucose oxidase enzyme; NiCo_2_N/N-GR core–shell—Nitrogen-doped graphene-encapsulated nickel cobalt nitride; Ni_2_Co_1_-BDC—Nickel cobalt bimetallic metal–organic framework; GR/CuO NPs—Copper oxide on graphene; CuNPs/rGO—Copper nanoparticles on graphene oxide; PtPd-IL-rGO—PtPd Nanoparticles/reduced graphene oxide composites; Ni(OH)_2_/3D GF—Nickel hydroxides on 3D graphene oxide; Ni(OH)_2_/rGO/MWCNTs/GCE—Ni(OH)_2_/Electroreduced graphene oxide-multiwalled carbon nanotube film modified glassy carbon electrode; PVP/GNs/NiNPs/CS—Polyvinylpyrrolidone–graphene nanosheets–nickel nanoparticles–chitosan nanocomposite; GOx—Glucose oxidase, Cs/Cu-MOF/SPCE—chitosan-based copper MOF onto a screen-printed carbon electrode; CuO@CNTFs—copper oxide-modified carbon nanotubes; PVP–TiO_2_ NCs—polyvinylpyrrolidone and titanium dioxide nanocomposites; MOF MnOx-CNFs—manganese-based metal–organic frameworks embedded in carbon nanofibers.

**Table 2:** Different hybrid composite based H_2_O_2_ sensors and their sensitivity, concentration detection range, and LOD.

Electrocatalyst Material	Linear Range	Sensitivity (μA·mM^−1^·cm^−2^)	LOD	Ref.
PAn-PAA/Au	0.04 to 12 mM	417.5	0.02 mM	[140]
Poly(CoTBImPc)/CNTs/GCE	10–100 nM	3.4522	2 nM	[141]
GO/poly(CoTBIPc)	2–200 μM	-	0.6 μM	[142]
Functionalization of hemoglobin (Hb) macromolecule on 3D structured Ni foam	50–850 μM	0.39	0.41 μM	[143]
Platinum–nickel decorated polyaniline nano-spheres on reduced graphene oxide (rGO/PANI@PtNi)	0.1–126.4 mM	-	0.5 μM	[146]
Cu_2_O/N graphene/GCE	5–357 μM	-	0.80 μM	[147]
rGO/carbon ceramic with CdS-hemoglobin	2–240 μM	1.056	0.24 μM	[148]
Carboxyl (COOH)-functionalized Graphene oxide	0.006–0.8 μM		1 nM	[149]
3D Porous CeO2/Reduced Graphene Oxide Xerogel Composite	60.7 nM to 3.0 μM	1.978	30.40 nM	[150]
Silver nanoparticles (AgNPs)/reduced graphene oxide (rGO) nanocomposites	5 to 620 μM	49	3.19 μM	[151]
PPy–Ag/Cu	1.0–37.0 mM	445.78	0.063 μM	[152]
Co_3_O_4_ nPTLS (featuring petal-shaped Co_3_O_4_ nanostructures)	25–5000 μM	201	5.2 µM	[153]
Copper-exchanged zeolite-modified electrode	Up to 30 mM	0.87	10 µM	[154]
Laser-Synthesized Pd/LIG Nanocomposite-Modified Screen-Printed Electrode	5 µM–0.9 mM	-	0.37 µM	[155]
Copper porphyrinic nanosheet decorated bismuth metal–organic framework modified electrode	10–120 μM	-	0.20 μM	[156]
Coumarin derivative compound(CTH)/MWCNT-modified GC electrode	50–400 nmol L^−1^	-	12 nmol	[157]
PtNPs/porous GR	1–1477 mM	341.14	0.50 μM	[158]
Freestanding graphene papers	1–30 mM	500,000	100 nM	[159]
Hollow TiO_2_-modified rGO microspheres	0.1–360 μM	417.6	10 nM	[160]
CuO/graphene nanosphere composite	0.01–0.1 mM	-	6.88 μM	[161]
Ni(OH)_2_/rGO/MWCNTs	10–9050 μM	2042.0	4.0 μM	[162]
Cytochrome c (Cyt c)/GO/CNT/AuNPs	0.001–0.14 mM	0.533	0.027 nm	[163]

Note: PAn-PAA/Au—polyaniline-polyacrylic acid composite on gold electrode; Poly(CoTBImPc)/CNTs/GCE—polymeric cobalt tetrabenzimidazole phthalocyanine on CNT-modified GCE; GO/poly(CoTBIPc)—cobalt tetraamine benzamide phthalocyanine on graphene oxide; Cu_2_O/N graphene/GCE—Cuprous oxide and nitrogen-doped graphene in a Nafion matrix; rGO/carbon ceramic with CdS-hemoglobin—Reduced graphene oxide/carbon ceramic electrode-modified with CdS hemoglobin; cytochrome c (Cyt c)/GO/CNT/AuNPs—cytochrome c immobilized on a graphene oxide–carbon nanotube composite; PPy–Ag/Cu—polypyrrole (PPy) electropolymerized on GCE, then Ag and Cu nanoparticles modified together on the surface of polypyrrole by electrodeposition.

**Table 3:** Various hybrid composite modified electrodes employed for UA sensors and their sensitivity, concentration, detection range, and LOD.

Electrocatalyst Material	Linear Range	Sensitivity (μA·mM^−1^·cm^−2^)	LOD	Ref.
Ti_3_C_2_T_x_/polypyrrole (MXene/PPy)	50–500 μM	-	0.15 μM	[164]
(BSA)/BLG-MWCNTs-PtNPs/GCE	0.02 to 0.5 mM	31.131	0.8 μΜ	[165]
PAH/UOx/PSS/(PAH/PSS)_2_	1–70 μM	10.64 × 10^−3^	0.81 μΜ	[166]
PolyPyrrol/α-Fe_2_O_3_	5–200 μM	-	1.349 μM	[167]
Cobalt-Copper bimetallic nanoclusters	2 to 1000 μM	-	0.61 μM	[168]
UOx/MWCNT-CMC/Au	20–5000 μΜ	0.233	2.8 μΜ	[169]
GA/UOx/Chitosan/SACNT/Pt	100–1000 μΜ	0.518	1.0 μΜ	[170]
Zinc hydroxide nitrate-sodium dodecylsulfatebispyribac modified with multiwalled carbon nanotube (ZHN-SDS-BP/MWCNT)	5.0 µM to 0.7 mM	-	0.371 μM	[171]
UOx/Fc/Cu_2_O/GCE	0.1–1000 μΜ	0.002	0.06 μΜ	[172]
Functionalized Aryl Derivative of Phenothiazineand PAMAM-Calix-Dendrimers	10 nM to 20 μM	-	4 nM	[173]
Graphite screen-printed electrodes/Prussian blue/poly(4-aminosalicylic acid)/uricase	10–200 μΜ	-	3.0 μΜ	[174]
Carboxyl functionalized multiwall carbon nanotubes (MWCNT-COOH)	0 to 1.6 mM	-	3.58 μM	[175]
Puffy balls-shaped cobalt oxide nanostructure-modified glassy carbon	0–1000 μM	-	2.4 μM	[176]

Note: Ti_3_C_2_T_x_/polypyrrole (MXene/PPy)—MXene-type Ti_3_C_2_T_x_ molecule with polypyrrole; (BSA)/BLG-MWCNTs-PtNPs/GCE—β-Lactoglobulin-functionalized multiwall carbon nanotubes; PAH/UOx/PSS/(PAH/PSS)_2_—uricase crowding via polyelectrolyte layers coacervation for carbon fiber; UOx/MWCNT-CMC/Au—uricase/carbon nanotube/carboxymethyl cellulose on gold electrode; GA/UOx/Chitosan/SACNT/Pt—super-aligned carbon nanotubes; UOx/Fc/Cu_2_O/GCE—nano-cube cuprous oxide/ferrocene/uricase-modified glassy carbon.

**Table 4:** Various hybrid composite modified electrodes employed as AA biosensors.

Electrocatalyst Material	Linear Range	LOD	Ref.
PDbS–rGO/SPCE	10–1100 µM	0.88 µM	[180]
Elecrosynthesized molecularly imprinted polypyrrole	30–2400 µM	21 µM	[181]
PEDOT/rGO/GCE	0.1–907 µM	1.5 µM	[182]
C_3_F_7_-azo-TaWO_6_/GCE	100–1000 µM	4.6 µM	[183]
(PdTAPc)/MWCNTs/GCE	3–24 µM	1 µM	[184]
CB-CNT/PI/GCE	100–5000 µM	75 µM	[185]
Co_3_O_4_(Cobalt oxide)	0.5 to 6.5 mM	0.001 mM	[186]
Cadmium sulfide–gold (CdS–Au) composite nanomaterials	0.01–200 μM	0.2 nM	[187]
GCE/MWCNT-CoTMBANAPc	7.5–70 nM	6.6 µM	[188]

Note: PDbS–rGO/SPCE—Block copolymer-dispersed graphene; 3D-MoS_2_/rGO/Au/GCE—gold nanoparticles on a 3D-network consisting of a MoS_2_/rGO nanocomposite; PEDOT/rGO/GCE—poly (3,4ethylene dioxythiophene)-anchored reduced graphene oxide nanocomposite; C_3_F_7_-azo-TaWO_6_/GCE—2D-titanium carbide (MXene); (PdTAPc)/MWCNTs/GCE—Palladium phthalocyanine composite with multiwalled carbon nanotubes; CB-CNT/PI—Carbon black–carbon nanotube co-doped polyimide; Nano SnO_2_/MWCNTs/CPE—SnO_2_ nanoparticles/multiwalled carbon nanotubes/carbon paste electrode; Pt-Gr-CNT—Pt-decorated graphene–carbon nanotubes; CoTMBANAPc—tetra8[(*E*)(4-methoxybenzylidene)amino] naphthalene1amine cobalt (II) phthalocyanine.

**Table 5:** Various macromolecules and their hybrid composites as DA biosensors.

Electrocatalyst Material	Linear Range	Sensitivity	LOD	Ref.
poly(luminol–benzidine sulfate)	1–20 nM	-	0.5 nM	[209]
AuMS	10–80 μM	-	1.28 nM	[210]
CQDs/CuO	1–180 μM	-	25.40 μM	[211]
PANI-NF/Pt	62.5–603 μM	-	33.30 μM	[212]
CAuNE	1–100 μM	-	5.83 μM	[213]
PEDOT-LSG	1–150 μM	0.220 μA/μM	0.33 μM	[214]
PGE/GNS/DA	1–100 ng/L	-	0.29 ng/L	[215]
MWCNT/MoS_2_/Co_3_O_4_ PHs	0.03–1950.2 μA 2150.2–5540 μM	2.197 μA/μM 3.486 μA/μM	0.013 μM	[216]
DA-RC	0.085–700 ng/mL	-	0.085 ng/mL	[217]
DAAPT-AuNPs	200 fM–20 nM	-	200 fM	[218]
Conjugated polymer P(NIPAAm149-stMAAmBO19) and P(LAEMA21),	1 nM–0.1 mM	-	5 × 10^−11^ mol L^−1^	[219]
Conducting polymers/nanofibers	1–100 nM	-	0.1 nM	[220]
Polypyrrole/MoO_3_ NP	5–1000 nM	-	2 nM	[221]
MWCNT-IE	0.5–10 μM	8.06 µA/μM	0.99 μM	[222]
Sol–gel CCE	0.5–50 μM	0.0414 μA/μM	0.07 μM	[223]
	0.5–20 μM	0.75 μA/μM	0.1 μM	[223]
CDP-GS-MWCNTs	0.15–21.65 μM	-	0.05 μM	[224]

Note: PdTAPc/MWCNT—Tetra-amino-substituted palladium phthalocyanine-modified multiwalled carbon nanotubes; Poly(CoTNBAPc)—Cobalt (II) tetra[β-N-(4-nitrophenyl) benzamide] phthalocyanine; poly(luminol–benzidine sulfate)-Poly(luminol-benzidine sulphate), AuMS—Gold nanoparticles intercalated mesoporous; CQDs/CuO—Carbon quantum dots/copper oxide; PANI-NF/Pt—Polyaniline nanofiber-modified platinum electrode; CAuNE—Cylindrical gold nanoelectrode; PEDOT-LSG—Poly(3,4-ethylenedioxythiophene)-modified laser/graphene; PGE/GNS/DA—Pencil graphite electrode gold nano-stars; MWCNT/MoS_2_/Co_3_O_4_ PHs—MWCNTs/MoS_2_ decorated cobalt oxide polyhedrons composite film-modified electrode; DA-RC—D(3) dopamine receptor; DAAPT-AuNPs—DA DNA aptamer gold nanoparticle conducting polymers/nanofibers; Polypyrrole/MoO_3_ NP—Polypyrrole/MoO_3_ nanoparticles; MWCNT-IE—Carbon nanotube intercalated graphite electrodes; Sol–gel CCE—Carbon ceramic electrode prepared by sol–gel technique; CDP-GS-MWCNTs—fraphene/poly-cyclodextrin/MWCNTs nanocomposite.

**Table 7:** Macromolecules and their hybrid-fabricated electrodes employed as cholesterol biosensors.

Electrocatalyst Material	Sensitivity	Linear Range	LOD	Ref.
PLE	1422.22 µA·mM^−1^·cm^−2^	625–9375 µM	-	[271]
PO/rGO	95.6 µA·mM^−1^·cm^−2^	100–2 × 10^4^ µM	1.02 µM	[272]
CNf/Polymer	226.30 µA·mM^−1^·cm^−2^	0.04–600 mg dL^−1^	0.002 mg dL^−1^	[273]
G/PVP/PANI/Paper	34.77 µA·mM^−1^·cm^−2^	50–10,000 µM	1 µM	[274]
PANI/MWCNTs/Starch	800 µA·mM^−1^·cm^−2^	32–5000 µM	10 µM	[275]
Graphene/β-CD	0.01 µA·mM^−1^·cm^−2^	0.001–0.10 mM	1 µM	[276]
Poly (CBNP)/PGE	1.49 µA·µM^−1^·cm^−2^	0.0025–0.0275 mM	0.0004 mM	[277]
Chitosan/silica/MWCNTs	1.55 µA·mM^−1^·cm^−2^	8 × 10^−6^–4.5 × 10^−4^ M	1 mM	[278]
PBNPs	2.1 µA·mM^−1^·cm^−2^	0–15 mM	0.2 mM	[279]
SPE/NPG	32.68 µA·mM^−1^·cm^−2^	50 µM to 6 mM	8.36 µM	[280]
GK-Pd NPs	–	5–100 µM	3.7 µM	[281]

Note: PLE—Pencil lead electrode; PO/rGO—Polyoxometalate/rGO composite; CNf/polymer—Carbon nanofiber/polymer nanocomposite; G/PVP/PANI/paper—Graphene/polyvinylpyrrolidone/polyaniline nanocomposite; PANI/MWCNTs/Starch—Polyaniline/multiwalled carbon nanotube/starch; Graphene/β-CD—β-cyclodextrin functionalized grapheme; Poly (CBNP)/PGE—Pencil graphite electrode/thiophene-3-boronic acid and 3-aminophenyl boronic; chitosan/silica/MWCNTs—Chitosan/silicas and multiwalled carbon nanotubes; PBNPs—Prussian blue nanoparticle;SPE/NPG— screen-printed electrode with nanoporous gold; GK-Pd NPs — gum kondagogu biopolymer palladium nanoparticles).

**Table 8:** Summary of the parameters for the sensing of cancer cells using different macromolecule-based hybrid composites.

Biomarkers	Electrocatalyst Material	Linear Range	LOD	Ref.
HER2-ECD	GE/RGONs/Rh-NPs	10–500 ng/mL	0.667 ng/mL	[303]
miRNA-21	NFG/AgNPs/PANI/RGo/Au	10 fM–10 µM	0.2 fM	[304]
CEA	GO-carbon nanotubes–hemin	1 fg/mL–0.01 µg/mL	0.82 fg/mL	[305]
miRNA-21	Graphene/GCE	10^−14^ to 10^−8^ M	3 × 10^−15^ M	[306]
CYFRA 21-1	Au layer + SAM (HDT) + AuNP + SAM (AHT) + anti-CYFRA 21-1 + anti-CYFRA 21-1/QD	-	0.1 ng/mL	[307]
CK7	Gold layer + cytokeratin 7 antibodies	-	0.4 nM	[308]
miRNA	Gr/poly-L-lysine	-	1 fM	[309]
M CF-7 cells	Apt/GO/AuNPs	10–10^5^ cells/mL	8 cells/mL	[310]
miRNA-21	Gr/AuNPs/PPY	1 fM–1 nM	0.02 fM	[311]
miRNA-21	GO/AuNPs/MgO	0.1–100 fM	50 aM	[312]
MicroRNA	Au film + DNA tetrahedron probes (DTPs)	-	0.8 fM	[313]
CA15-3	Ag-rGO/AuNPs	15–125 U/mL	15 U.mL^−1^	[314]
PSA	CNT/Au-Ab1/Ag/Ab2-HRP	4–10 ng/mL	0.5 pg/mL	[315]

Note: GE/RGONs/Rh-NPs—Reduced graphene oxide nanosheets and rhodium nanoparticles on the graphite electrode surface; NFG/AgNPs/PANI/RGo/Au—Nitrogen-doped functionalized graphene/silver nanoparticles/polyaniline/gold; GO-carbon nanotubes–hemin—Hemin, Graphene oxide, and multiwalled carbon nanotubes; Graphene/GCE—Graphene modified glassy carbon electrode; Au layer + SAM (HDT) + AuNP + SAM (AHT) + anti-CYFRA 21-1 + anti-CYFRA 21-1/QD—Antibody-quantum dot/ꭤ-fetoprotein/carcinoembryonic antigen/cytokeratin fragment; Gr/poly-L-lysine—poly-L-lysine-functionalized graphene field; Apt/GO/AuNPs—PolydA-aptamer functionalized gold nanoparticle/graphene oxide; Gr/AuNPs/PPY—Gold nanoparticles/graphene/polypyrrole composite; GO/AuNPs/MgO—Graphene oxide/gold nanoparticles/magnesium oxide; Au film + DNA tetrahedron probes (DTPs)—Gold film/DNA tetrahedron probes; Ag-rGO/AuNPs—Silver nanoparticle-reduced graphene oxide/Gold nanoparticles; CNT/Au-Ab1/Ag/Ab2-HRP—Gold nanoparticle film electrode and multienzyme particle.

## Data Availability

Data are contained within the article.
